# Extracellular nucleases and extracellular DNA play important roles in *Vibrio cholerae* biofilm formation

**DOI:** 10.1111/j.1365-2958.2011.07867.x

**Published:** 2011-10-27

**Authors:** Andrea Seper, Vera H I Fengler, Sandro Roier, Heimo Wolinski, Sepp D Kohlwein, Anne L Bishop, Andrew Camilli, Joachim Reidl, Stefan Schild

**Affiliations:** 1 Institut fuer Molekulare Biowissenschaften, Karl-Franzens-Universitaet GrazHumboldtstrasse 50, 8010 Graz, Austria; 2 Howard Hughes Medical Institute and the Department of Molecular Biology and Microbiology, Tufts University School of MedicineBoston, MA 02111, USA

## Abstract

Biofilms are a preferred mode of survival for many microorganisms including *Vibrio cholerae*, the causative agent of the severe secretory diarrhoeal disease cholera. The ability of the facultative human pathogen *V. cholerae* to form biofilms is a key factor for persistence in aquatic ecosystems and biofilms act as a source for new outbreaks. Thus, a better understanding of biofilm formation and transmission of *V. cholerae* is an important target to control the disease. So far the *Vibrio* exopolysaccharide was the only known constituent of the biofilm matrix. In this study we identify and characterize extracellular DNA as a component of the *Vibrio* biofilm matrix. Furthermore, we show that extracellular DNA is modulated and controlled by the two extracellular nucleases Dns and Xds. Our results indicate that extracellular DNA and the extracellular nucleases are involved in diverse processes including the development of a typical biofilm architecture, nutrient acquisition, detachment from biofilms and the colonization fitness of biofilm clumps after ingestion by the host. This study provides new insights into biofilm development and transmission of biofilm-derived *V. cholerae*.

## Introduction

The causative agent of cholera is the Gram-negative bacterium *Vibrio cholerae* (Koch, 1884). The intestinal disease is characterized by profuse secretory diarrhoea and vomiting that rapidly leads to dehydration and death by hypovolaemic shock. The recent outbreak of cholera in Haiti has drawn public attention, but it should be emphasized that cholera is currently endemic in approximately 50 countries and *V. cholerae* infects several million individuals globally each year (Sack *et al*., 2004; WHO, 2009; Ryan, 2011). Hallmarks of the life cycle of the clinically relevant *V. cholerae* strains are the transitions between two dissimilar habitats: as a natural inhabitant of the aquatic ecosystems and as a pathogen in the human gastrointestinal tract (Reidl and Klose, 2002; Schild *et al*., 2008a; Nelson *et al*., 2009).

Finally, a better understanding of the environmental persistence and survival of *V. cholerae* has become a goal for possible control of the spread of the disease (Islam *et al*., 1993; Colwell, 1996; Sack *et al*., 2004). One key factor for environmental survival and transmission of bacteria is the ability to form matrix-enclosed surface-associated communities, also called biofilms. In the aquatic environment *V. cholerae* is believed to form biofilms on surfaces provided by plants, algae, zooplankton, crustaceans and insects (Huq *et al*., 1990; Tamplin *et al*., 1990; Colwell, 1996). In particular chitin, one of the most abundant biopolymers in the aquatic environments, is an important substrate for *V. cholerae* and has impact on its physiology including the utilization as a carbon and nitrogen source as well as an inducer of natural competence (Meibom *et al*., 2004; 2005). Upon oral ingestion by its human host, *V. cholerae* cells associated in biofilms might be protected from digestive enzymes, acidic pH and antimicrobial substances, thereby enhancing colonization and facilitating transmission of the disease (Zhu and Mekalanos, 2003; Huq *et al*., 2008). However, it has been shown that even if the natural biofilm structure is mechanically dispersed the resulting *V. cholerae* cells greatly out-compete planktonically grown cells in the infant mouse model (Tamayo *et al*., 2010). Thus, biofilm formation seems to play a major role in the physiology, ecology and epidemiology of *V. cholerae*.

Recent studies analysed structural prerequisites for *V. cholerae* biofilm formation including flagella, pili and exopolysaccharide biosynthesis (Watnick *et al*., 1999; 2001; Watnick and Kolter, 1999; Chiavelli *et al*., 2001; Lauriano *et al*., 2004; Meibom *et al*., 2004; Moorthy and Watnick, 2004; 2005; Reguera and Kolter, 2005; Yang *et al*., 2009). In addition, signalling mechanisms including two-component systems, quorum sensing and c-di-GMP involved in regulation of biofilm formation have been elucidated (Tischler and Camilli, 2004; 2005; Houot *et al*., 2010; Krasteva *et al*., 2010). So far, the *V. cholerae* exopolysaccharide (VPS) is the only characterized matrix component required for biofilm formation in most *V. cholerae* isolates analysed (Yildiz and Schoolnik, 1999; Kierek and Watnick, 2003; Yildiz *et al*., 2004). The genes encoding proteins for VPS synthesis and secretion are arranged in two clusters *vpsA-K* (VC0917-27) and *vpsL-Q* (VC0934-9). The UhpA family regulator VpsT and the response regulator VpsR positively regulate transcription of *vps* genes (Yildiz *et al*., 2001; Casper-Lindley and Yildiz, 2004). Furthermore, HapR, a key regulator of the quorum sensing cascade in *V. cholerae*, acts as a negative regulator of biofilm formation by repression of *vps* genes including *vpsT* and *vpsR* (Yildiz *et al*., 2004; Beyhan *et al*., 2007; Waters *et al*., 2008). Because transcription of *hapR* is controlled by quorum sensing and RpoS, biofilm formation seems to be regulated by central physiological signals, like cell density or carbon concentration (Hammer and Bassler, 2003; Zhu and Mekalanos, 2003; Yildiz *et al*., 2004).

In general, the matrix of bacterial biofilms primarily consists of exopolysaccharides, but compounds like proteins, lipids and nucleic acids can be found in biofilms of microorganisms and serve important functions (Goller and Romeo, 2008; Karatan and Watnick, 2009; Flemming and Wingender, 2010). Recently, extracellular DNA (eDNA) was shown to be required for biofilm formation in *Pseudomonas aeruginosa* (Whitchurch *et al*., 2002). Since then, eDNA has been found in biofilms of several Gram-positive and Gram-negative bacteria such as *Streptococcus* ssp., *Enterococcus faecalis*, *Listeria monocytogenes, Neisseria mengitidis* and *Helicobacter pylori* (Moscoso *et al*., 2006; Thomas *et al*., 2008; Grande *et al*., 2010; Harmsen *et al*., 2010; Lappann *et al*., 2010). In almost every one of these studies a different role of eDNA in biofilms has been reported. Extracellular DNA is implicated to mediate initial attachment, act as a structural component stabilizing the biofilm matrix or is used as a nutrient source. To the contrary, in *Caulobacter crescentus* eDNA can bind and mask the polar holdfast of swarmer cells thereby inhibiting their attachment to biofilms (Berne *et al*., 2010). In *Bordetella* eDNA is important for maintaining biofilm integrity *in vitro* and *in vivo* (Conover *et al*., 2011). Thus, the physiological roles of eDNA in biofilms seem to be versatile, although still poorly characterized how it is regulated as a component of the biofilm.

*Vibrio cholerae* encodes two extracellular nucleases Dns (VC0470) and Xds (VC2621) that are secreted into the culture supernatant (Newland *et al*., 1985; Focareta and Manning, 1987; 1991a,b). Deletion of both extracellular nucleases, especially Dns, increases the transformation efficiency regardless of whether the competence of *V. cholerae* is induced by growth on chitin or CaCl_2_-treatment (Focareta and Manning, 1991a; Blokesch and Schoolnik, 2008). This is probably due to increased stability of exogenous DNA in the absence of the nucleases. Xds is a 100 kDa polypeptide and is assigned by computational analysis to the protein family PF03372, which includes a large number of Mg^2+^-dependent endonucleases and exonucleases (Mol *et al*., 1995; Dlakic, 2000). Additionally, a recent study identified *xds* as a gene induced at a late stage of infection in the infant mouse small intestine (Schild *et al*., 2007). Dns, also known as VcEndA, has been crystallized and comprehensively analysed for its biochemical properties (Altermark *et al*., 2007; Niiranen *et al*., 2008). It belongs to the endonuclease I superfamily and consequently should cleave nucleic acids at non-specific internal sites. Recently, it has been demonstrated that *dns* is repressed by HapR and therefore co-regulated with the *vps* genes (Blokesch and Schoolnik, 2008).

In this study we show that deletion of Xds and Dns results in increased biofilm formation. Intrigued by this observation, we identified and characterized eDNA as a constituent of *V. cholerae* biofilms. Our data demonstrate that the extracellular nucleases control the level of eDNA and are involved in multiple processes including the development of a typical three-dimensional biofilm structure, detachment from a mature biofilm and utilization of eDNA as a nutrient source. Infection studies indicate that the dissolution of biofilms driven by the activity of the nucleases is a crucial step for the colonization fitness of *V. cholerae*.

## Results

### Deletion of *dns* and/or *xds* results in an increase of biofilm formation

**Fig. 1:**
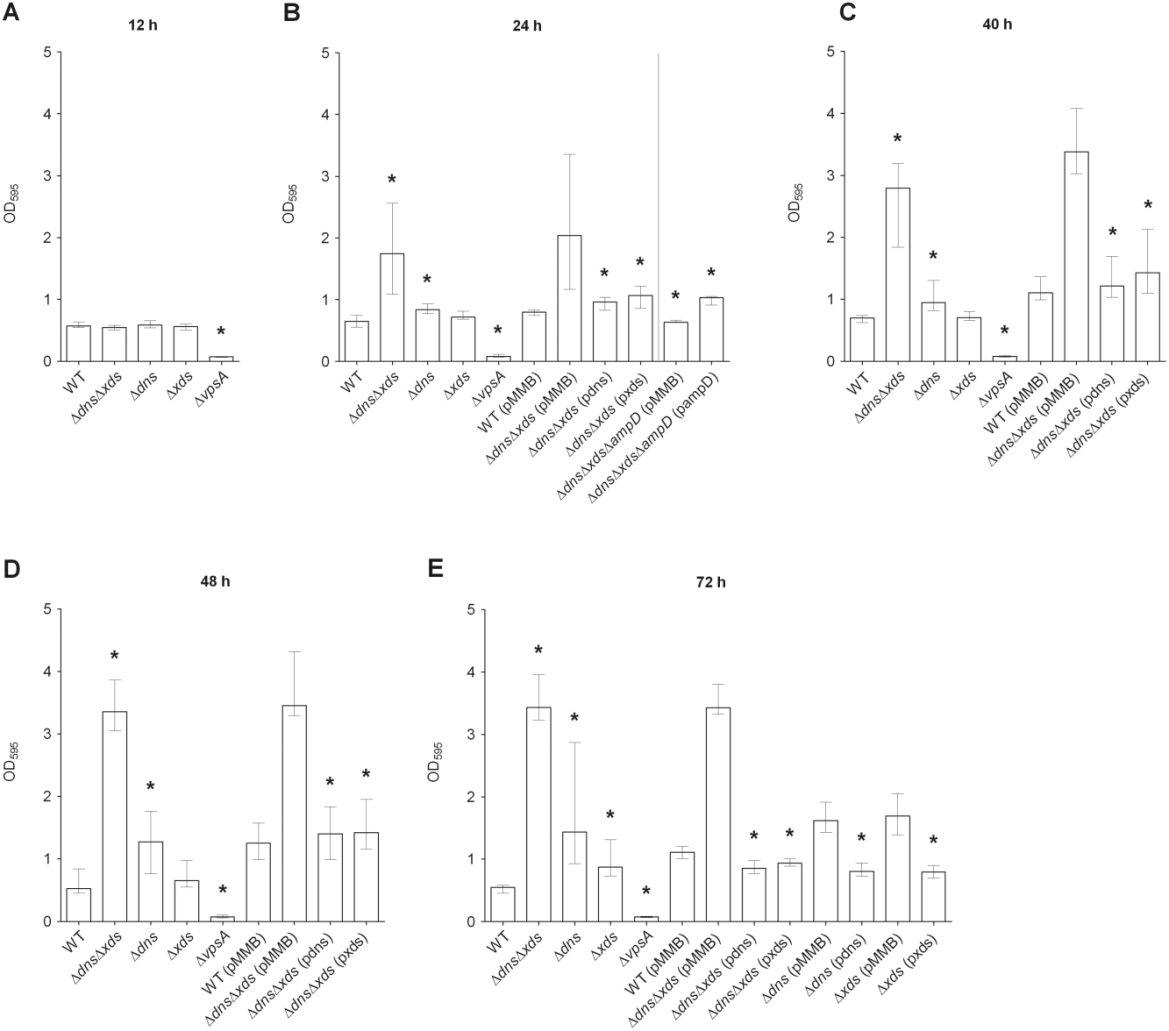
Deletion of *dns* and *xds* results in an increase of biofilm formation. Biofilms of the wild type (WT), deletion mutants, mutants and WT with empty pMMB plasmid or complemented mutants, as indicated, were quantified after 12 h (A), 24 h (B), 40 h (C), 48 h (D) and 72 h (E). The time points are indicated on top of each panel. The biofilm formation capacity was assayed under static conditions by crystal violet staining and subsequent determination of the OD_595_. Shown are the medians from at least eight independent measurements. The error bars indicate the interquartile range. Significant differences (*P < 0.05) are indicated for the following comparisons: deletion mutants with wild type; Δ*dns*Δ*xds* (pdns), Δ*dns*Δ*xds* (pxds) and Δ*dns*Δ*xds*Δ*ampD* (pMMB) with Δ*dns*Δ*xds* (pMMB); Δ*dns*Δ*xds*Δ*ampD* (pMMB) with Δ*dns*Δ*xds*Δ*ampD* (pampD); Δ*dns* (pdns) with Δ*dns* (pMMB); and Δ*xds* (pxds) with Δ*xds* (pMMB).

To determine whether nucleases play a role in *V. cholerae* biofilm formation, in-frame deletion mutants of *dns* and *xds* as well as a double deletion mutant were generated. Their biofilm formation capacity was investigated after 12, 24, 40, 48 and 72 h using the static biofilm assay with crystal violet staining (Fig. 1). The VPS-deficient deletion mutant Δ*vpsA* served as negative control, as it is incapable of biofilm formation under static conditions (Yildiz and Schoolnik, 1999; Fong *et al*., 2010). At 12 h no significant difference between wild type and mutants was observed (Fig. 1A). After 24 h the Δ*dns*Δ*xds* mutant showed a significant twofold increase in biofilm formation compared with the wild type (Fig. 1B). Within the next days the biofilm amount of the double mutant further increased, while the wild type biofilm remained at an almost constant level (Fig. 1A–E). Accordingly, the differences in biofilm production were even more pronounced after 48 and 72 h where the Δ*dns*Δ*xds* mutant exhibited sixfold higher biofilm levels compared with the wild type (Fig. 1D and E). A single deletion of *xds* did not result in enhanced biofilm formation within the first 48 h, whereas deletion of *dns* showed a small, but significant increase in biofilm mass compared with the wild type within this period (Fig. 1A–D). At 72 h both single mutants exhibited significantly higher biofilm levels than the wild type (Fig. 1E). However, even at 72 h neither of the single mutants produced such large biofilm amounts as the double mutant. These data show that deletion of one extracellular nuclease can be partially compensated by the remaining nuclease in these biofilm assays. The growth rates of planktonic cells of Δ*dns*, Δ*xds* and Δ*dns*Δ*xds* mutants were similar to that of wild type (Fig. S1A). Thus, the increased biofilm formation capacity of the mutants is not the result of altered growth rates.

Wild type biofilm levels could be restored in the Δ*dns*Δ*xds* mutant for all relevant time points by the expression of *dns* or *xds in trans*, but not by the plasmid vector alone (Fig. 1B–E). Complementation was also achieved for the single mutants at 72 h by the expression of *dns* or *xds in trans* respectively (Fig. 1E). It should be noted, that the presence of the expression vector at later time points generally resulted in a slight increase of biofilm production. Similar observations have been previously reported for conjugative plasmids (Ghigo, 2001; Reisner *et al*., 2006). Because this was true for wild type as well as for the mutants it has to be a general effect and we therefore included vector control groups for all complementation analyses in this study. Taken together, these results demonstrate that absence of the two extracellular nucleases Dns and Xds results in increased amounts of the biofilm mass.

### The *dns* and *xds* genes are important for the development of the biofilm structure

**Fig. 2:**
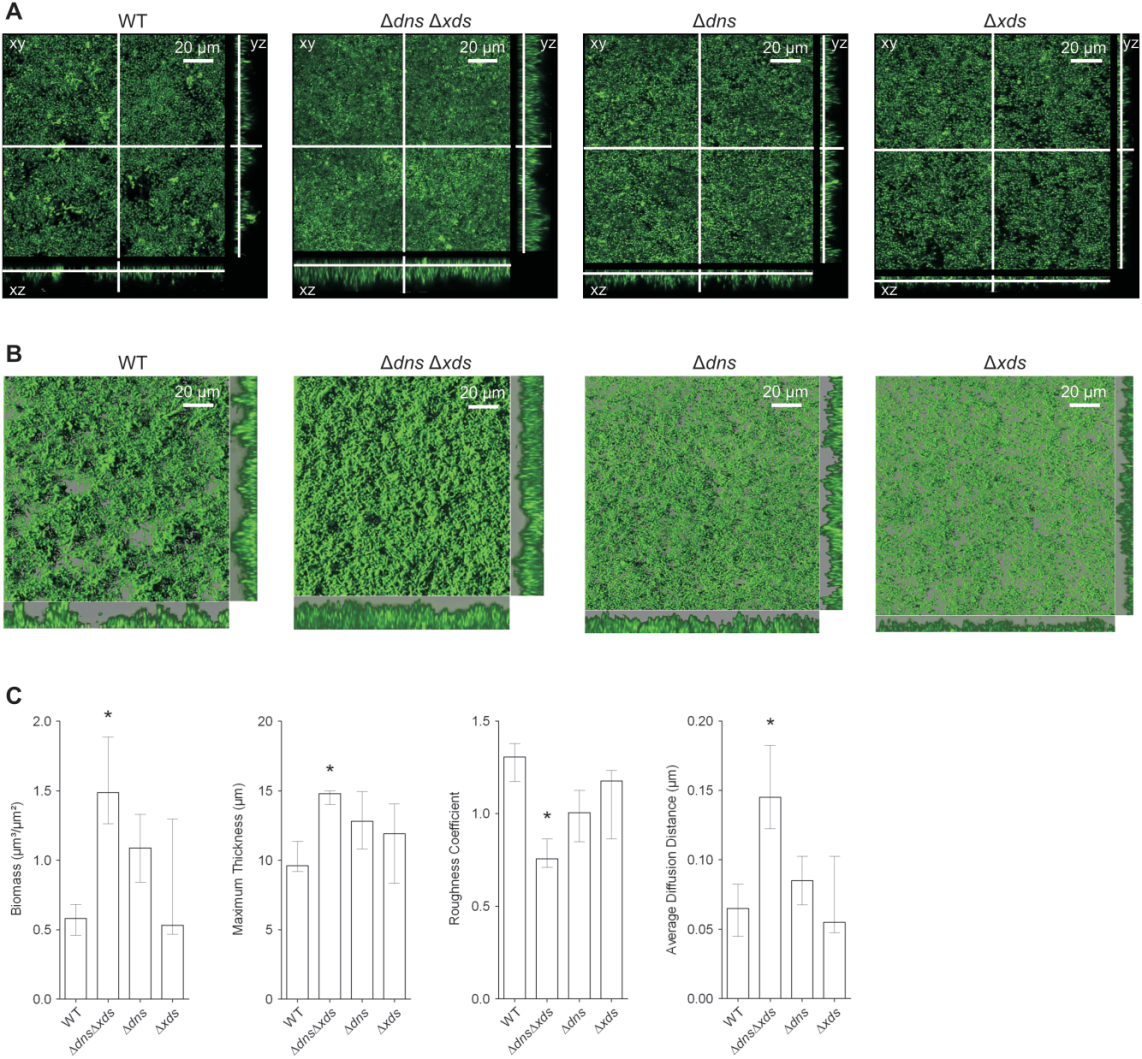
Absence of extracellular nucleases results in alterations of the biofilm architecture. A. Shown are confocal laser scanning microscopy images of the wild type, Δ*dns*Δ*xds*, Δ*dns* and Δ*xds* mutant biofilms as horizontal (xy) and vertical (xz and yz) projections (large and side panels, respectively). Biofilms were allowed to form for 9 h in flow cell chambers supplied with LB and stained with SYTO 9 fluorescent nucleic acid stain. Large panels represent selected single optical sections through the acquired three-dimensional data sets. B. Micrographs represent three-dimensional images of the wild type, Δ*dns*Δ*xds*, Δ*dns* and Δ*xds* mutant biofilms analysed by the IMARIS software package using same data sets as in panel (A). The large images are three-dimensional top-down images of the biofilms, and the small images to the right of and below the large images are side views of sections. Movies of the wild type and Δ*dns*Δ*xds* mutant biofilms, which allow views from different angles, are provided in the supporting information (bf_wt.mov and bf_mut.mov). C. Image stacks of the wild type and mutant biofilms were analysed for the biomass, the maximum thickness, the roughness coefficient and the average diffusion distance using the COMSTAT software. Shown are the medians of at least six image stacks from three independent experiments for each strain. The error bars indicate the interquartile range. Significant differences (*P < 0.05) of structural parameters are indicated for the multiple comparisons of the deletion mutants with the wild type.

We hypothesized that the increase of biofilm levels in the double mutant might also affect a natural biofilm development and the biofilm architecture. Therefore, the three-dimensional structure of wild type and mutant biofilms were analysed using a flow cell system. Initial attachment was investigated after incubation for 2 h in Luria–Bertani (LB) or 50-fold diluted LB (2%), while flow cell biofilms were grown for 9 or 24 h using undiluted LB or 2% LB broth respectively. Bacteria were stained with the green fluorescent stain SYTO 9 and visualized by confocal laser scanning microscopy (Figs 2, S2 and S3). Quantitative analysis of these biofilm images was performed with COMSTAT (http://www.comstat.dk) (Heydorn *et al*., 2000; M. Vorregaard *et al*., pers. comm.). At the early 2 h time point no difference in the surface coverage of the wild type and the Δ*dns*Δ*xds* mutant was observed for both nutrient conditions investigated (Fig. S2). Thus, the Δ*dns*Δ*xds* mutant showed no obvious change during initial attachment. At the later time point, the wild type biofilm showed the characteristic architecture of a mature three-dimensional biofilm with pillars of cells separated by fluid-filled channels as previously described by others (Fig. 2) (Watnick and Kolter, 1999; Watnick *et al*., 2001; Yildiz *et al*., 2004; Yildiz and Visick, 2009). In contrast, the biofilm of the Δ*dns*Δ*xds* mutant appeared to be very thick and compact, covering the entire surface of the flow cell without any visible fluid-filled channels (Fig. 2). The differences in the structural parameters of the biofilms were also confirmed by quantitative analysis using COMSTAT, which revealed a significant threefold increase in biomass as well as a significant 30% greater maximum thickness of the Δ*dns*Δ*xds* mutant biofilms compared with the wild type (Fig. 2C). Additionally, the roughness and average diffusion distance were analysed (Fig. 2C). The biofilm roughness provides a measure for the thickness variation of the biofilm and is an indicator for biofilm heterogeneity (Heydorn *et al*., 2000). The average diffusion distance indicates the shortest average distance from a pixel containing biomass to a pixel without biomass. This is an indicator for distances over which nutrients and substrate components have to diffuse to the bacteria (Yang *et al*., 2000). The Δ*dns*Δ*xds* mutant biofilms exhibited a significant lower roughness, but a significant higher average diffusion distance compared with the wild type. Similar results were obtained using 2% LB broth (Fig. S3). These results confirmed that the biofilm architecture of the Δ*dns*Δ*xds* mutant showed less heterogeneity in combination with higher density. Hence, the double mutant biofilm lacked the typical structural details present in wild type biofilms. The biofilm morphology and structural parameters of the single mutants ranged between the wild type and the double mutant, strengthening the idea that the loss of one extracellular nuclease can be partially compensated by the remaining nuclease (Figs 2 and S3). Overall, these data suggest that the deletion of *dns* and *xds* leads to an uncoordinated accumulation of large amounts of biomass and formation of an unstructured biofilm.

### The increase of biofilm formation of the Δ*dns*Δ*xds* mutant is not related to *vps* expression

**Fig. 3:**
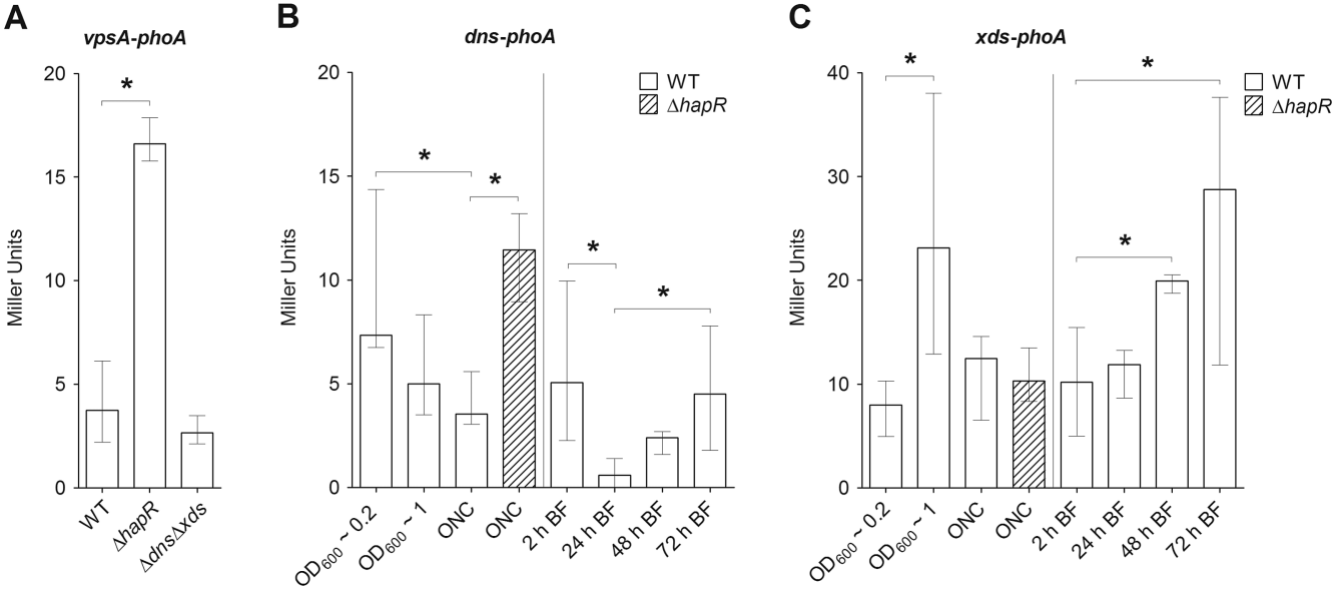
*vpsA* transcription is not altered in Δ*dnsΔxds* mutant and analysis of the *xds* and *dns* expression patterns. A. Alkaline phosphatase activities (in Miller Units) were measured from overnight cultures of wild type, Δ*hapR* mutant and Δ*dns*Δ*xds* mutant with a chromosomal *vpsA*-*phoA* transcriptional fusion. Shown are the medians from at least eight independent measurements. The error bars indicate the interquartile range. The activity in the Δ*hapR* mutant compared with the wild type is significantly different (*P < 0.05). B and C. Shown are the alkaline phosphatase activities (in Miller Units) of wild type (open bars) and Δ*hapR* mutant (shaded bars) with either a chromosomal *dns*-*phoA* (B) or *xds*-*phoA* (C) transcriptional fusion. Activities were measured from cultures grown with aeration until they reached an OD_600_ of ∼0.2 or ∼1, from overnight cultures (ONC) or from biofilms (BF) grown under static conditions at the indicated time points. Results in panels (B) and (C) are the medians from at least eight independent measurements. The error bars indicate the interquartile range. Significant differences (*P < 0.05) of the alkaline phosphatase activities are indicated for the respective single or multiple comparisons: Activities in the overnight cultures of the wild type and Δ*hapR* mutant carrying the *dns*-*phoA* or *xds*-*phoA* fusion; as well as biofilms of the wild type carrying the *dns*-*phoA* or *xds*-*phoA* at the different time points.

*Vibrio cholerae* mutants exhibiting increased biofilm formation compared with the wild type have been reported previously. However, these phenotypes correlated with enhanced *vps* gene expression and increased VPS production, as demonstrated for *hapR* mutants, rugose phase variants or mutants with increased c-di-GMP levels (Yildiz *et al*., 2001; Bomchil *et al*., 2003; Zhu and Mekalanos, 2003; Tischler and Camilli, 2004). To reveal whether the enhanced biofilm formation observed in the Δ*dns*Δ*xds* mutant was dependent on *vps* expression, chromosomal transcriptional fusions of a promoterless *phoA* reporter gene to *vpsA*, the first gene in the *vps-*I locus, were constructed in the wild type, the Δ*dns*Δ*xds* and the Δ*hapR* mutant. Thus, the measured PhoA activity reflects the transcription levels of *vpsA* in the respective strains. Because HapR acts as a negative regulator on *vps* genes, the Δ*hapR* mutants served as control for high *vps* gene expression (Hammer and Bassler, 2003; Zhu and Mekalanos, 2003; Yildiz *et al*., 2004). As expected, the Δ*hapR* mutant showed a significant fourfold increase in *vpsA-phoA* expression compared with the wild type (Fig. 3A). In contrast, Δ*dns*Δ*xds* mutant and wild type exhibited comparable levels of PhoA activity indicating similar transcription levels of *vpsA* in these strains. Consequently, *vps* transcription is not altered in Δ*dns*Δ*xds* mutant and the increased biofilm formation of Δ*dns*Δ*xds* cannot be simply explained by higher *vps* expression. Additionally, Δ*dns*, Δ*xds* and Δ*dns*Δ*xds* mutants showed no obvious changes in colony morphology, which are frequently observed in mutants with enhanced VPS production (Yildiz and Schoolnik, 1999; Watnick *et al*., 2001; Lauriano *et al*., 2004).

### *dns* and *xds* exhibit different expression patterns

To analyse the expression of *dns* and *xds*, chromosomal transcriptional fusions of a promoterless *phoA* reporter gene to *xds* and *dns* were constructed. PhoA activities during planktonic growth were measured from wild type carrying the *dns-phoA* or *xds-phoA* transcriptional fusion grown in LB with aeration until the culture reached an OD_600_ of ∼ 0.2 or ∼ 1 as well as from overnight cultures respectively (Fig. 3B and C). These time points reflect the early and late exponential phase as well as late stationary phase of a culture. As shown in Fig. 3B the highest activity for *dns-phoA* was detected directly in the early stage of the culture at the lowest cell density (OD_600_ ∼ 0.2). From there the expression steadily declined, which inversely correlates with the increase in cell density and accumulation of quorum sensing signals. This is consistent with a previous report, demonstrating that the quorum sensing regulator HapR is not only important for the repression of *vps* genes, but additionally acts as a negative regulator for *dns* transcription (Blokesch and Schoolnik, 2008). We verified that by measuring the activities of the *dns-phoA* fusion in a Δ*hapR* mutant. As expected, the expression of *dns-phoA* in overnight cultures is higher in the Δ*hapR* mutant indicated by the threefold increase of PhoA activity compared with the respective activity of the wild type (Fig. 3B).

In contrast, *xds* revealed a different expression pattern. The PhoA activities of the wild type carrying the *xds-phoA* transcriptional fusion peaked at an OD_600_ of ∼ 1, with lower levels at earlier and later stages of the culture (Fig. 3C). This already argues that regulation of *xds* is not strongly linked to the cell density and quorum sensing. To validate if *xds* expression is not influenced by HapR, the transcriptional *phoA*-fusion to *xds* was also constructed in a Δ*hapR* strain. Again, PhoA activities were measured from overnight cultures to allow accumulation of quorum sensing signals. In contrast to *dns-phoA*, comparable levels of PhoA activity in overnight cultures of the wild type and Δ*hapR* mutant carrying the *xds-phoA* transcriptional fusion were observed. Thus, expression of *xds* is independent of HapR and not co-regulated with *dns* via the quorum sensing cascade.

Furthermore, we used the wild type carrying the *dns-phoA* or *xds-phoA* transcriptional fusion to analyse the expression of both nucleases at different time points along the maturation of static biofilms (Fig. 3B and C). Expression of *dns-phoA* was relatively high at the 2 and 72 h time point reflecting very early and late stages of the biofilm development. In between expression levels first significantly dropped and then significantly increased again with the minimum level at 24 h. In contrast, expression of *xds-phoA* showed a steady increase in expression levels along the biofilm development, with the activities of the early 2 h and the late 48 and 72 h time points being significantly different. Thus, *dns* and *xds* exhibit different temporal expression patterns during biofilm formation. Furthermore, these results suggest that expression of *xds* and *dns* in biofilms and in the planktonic stage are not completely comparable and diverse environmental signals might influence the regulation of the extracellular nucleases depending on the respective condition.

### Dns and Xds exhibit distinct nuclease activities

Nuclease assays have been used to analyse and confirm the extracellular nuclease activity of Xds and Dns as described previously (Newland *et al*., 1985; Focareta and Manning, 1987; 1991a,b; Blokesch and Schoolnik, 2008). The wild type, the Δ*dns*Δ*xds* mutant and the Δ*dns*Δ*xds* mutant complemented with either *dns* or *xds* were analysed on DNase test agar plates (Fig. S4A). Degradation of DNA embedded in the agar is visualized by the zones of clearing for the wild type as well as for the Δ*dns*Δ*xds* mutant expressing Xds or Dns *in trans* respectively. In contrast, the Δ*dns*Δ*xds* mutant showed no detectable degradation of DNA. Furthermore, incubation of *V. cholerae* chromosomal DNA with culture supernatants from the respective strains and subsequent analysis of the degradation allowed visualization of the extracellular nuclease activities (Fig. S4B). Consistently, degradation of the chromosomal DNA was observed for the wild type and the Δ*dns*Δ*xds* mutant expressing Xds or Dns *in trans*, but not for the Δ*dns*Δ*xds* mutant with the vector control. It should be noted, that the impact of the two extracellular nucleases differs between the assays. Xds exhibits strong activity on the DNase test agar (Fig. S4A), while Dns seems to be the dominant enzyme in the second nuclease assay (Fig. S4B).

**Fig. 4:**
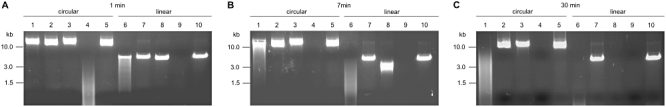
Xds and Dns exhibit different extracellular nuclease activities. Supernatants derived from bacterial cultures were assayed for their DNase activity by adding either circular (lanes 1 to 5) or linearized plasmid DNA (lanes 6 to 10). The supernatants were derived from following bacterial cultures: wild type (pMMB) (lane 1 and 6), Δ*dns*Δ*xds* (pMMB) (lane 2 and 7), Δ*dns*Δ*xds* (pxds) (lane 3 and 8), Δ*dns*Δ*xds* (pdns) (lane 4 and 9), LB broth control (lane 5 and 10). After incubation for 1, 7 and 30 min (panels A, B and C, respectively) the quality of the DNA was visualized on agarose gels. The incubation time is indicated on top of each panel.

To investigate the activities of Dns and Xds in more detail, we tested the nuclease activities of culture supernatants via degradation of circular or linearized DNA of the plasmid pBAD24 over 30 min (Fig. 4A–C). Degradation of linearized DNA by the wild type supernatant was already visible after 1 min, while the degradation for the circular DNA was delayed by a few minutes (Fig. 4A–C, lanes 1 and 6). Circular plasmid DNA was almost completely degraded after 30 min incubation (Fig. 4C, lane 1). In case of the LB control or the supernatant of the Δ*dns*Δ*xds* mutant no degradation was observable throughout the experiment regardless what type of DNA was used (Fig. 4A–C, lanes 5 and 10 or 2 and 7, respectively). Thus, the observed nuclease activity of the wild type supernatant correlates with presence of Dns and Xds, allowing degradation of circular or linearized DNA. This also indicates that Xds is not surface associated but rather secreted, as has already been shown for Dns by others (Focareta and Manning, 1987; Blokesch and Schoolnik, 2008).

By expression of each nuclease *in trans* in the Δ*dns*Δ*xds* mutant background the activities of Xds and Dns could be analysed separately. Rapid degradation of circular and linearized plasmid DNA was observed in the presence of Dns (Fig. 4A, lanes 4 and 9). In contrast, Xds was only capable of degrading linearized DNA (Fig. 4B/C, lanes 3 and 8). Complete degradation of linear DNA by Xds took notably longer compared with Dns. Furthermore, degradation of the linearized DNA by Xds occurred via an intermediate of defined decreased length, indicated by a relative distinct degradation band (Fig. 4B, lane 8), which migrates faster compared with the control (Fig. 4B, lane 10). In contrast, degradation by Dns resulted in a typical smear of the DNA. These assays allowed the discrimination of the enzymatic activities and suggest that Xds degrades only linearized DNA from its ends and thus is an exonuclease, whereas Dns degrades circular and linearized DNA into different sized products and thus is an endonuclease.

### Extracellular DNA is a component of the *V. cholerae* biofilm matrix

**Fig. 5:**
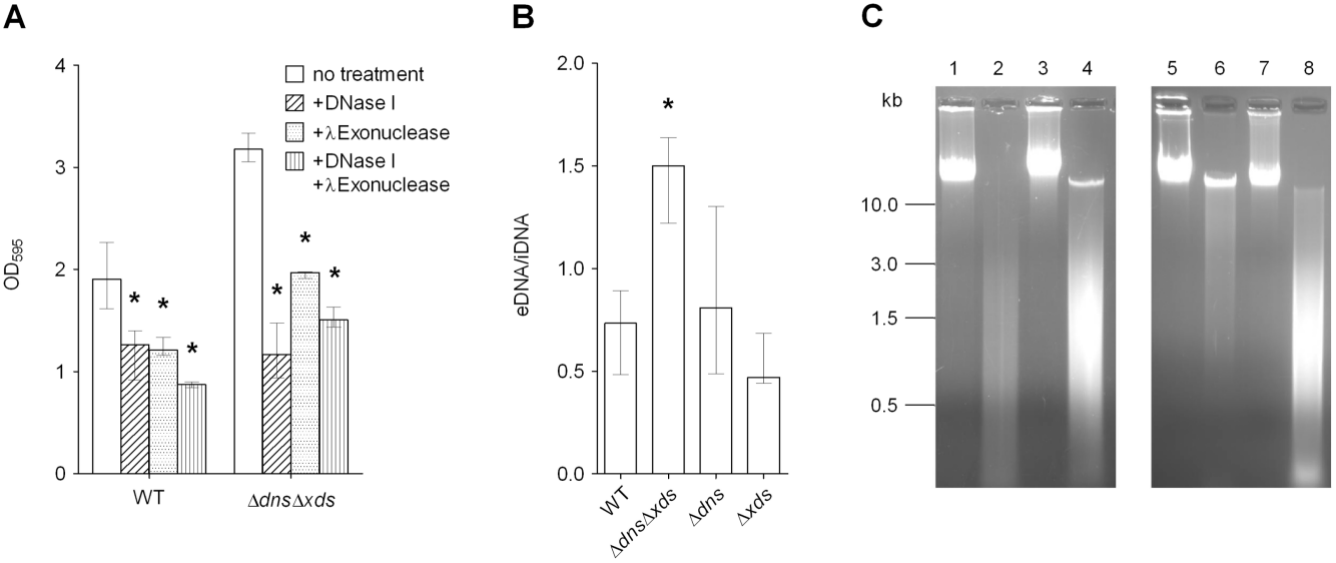
Extracellular DNA is a component of the *V. cholerae* biofilm matrix. A. Analysis of nuclease sensitivity of wild type and Δ*dns*Δ*xds* mutant biofilms. Biofilms grown for 21 h under static conditions were treated 3 h with DNase I (diagonally shaded bars), λExonuclease (dotted bars) or with a combination of DNase I and λExonuclease (horizontally shaded bars). Biofilm mass remaining after nuclease treatment was quantified by crystal violet staining. Significant differences compared with untreated biofilms of the wild type or Δ*dns*Δ*xds* mutant are indicated (*P < 0.05). B. Quantification of eDNA from wild type, Δ*dns*Δ*xds*, Δ*dns* and Δ*xds* mutant biofilms grown under static conditions. The bars represent the ratio eDNA to intracellular DNA (iDNA), which is significantly increased for the Δ*dns*Δ*xds* mutant compared with the wild type (*P < 0.05). Results of panel (A) and (B) are the medians from at least six independent measurements. The error bars indicate the interquartile range. C. Visualization of the quality of eDNA and iDNA derived from wild type, Δ*dns*Δ*xds*, Δ*dns* and Δ*xds* mutant biofilms by agarose gel analysis. Shown are representative samples of iDNA derived from wild type biofilm (lane 1), eDNA derived from wild type biofilm (lane 2), iDNA derived from Δ*dns*Δ*xds* mutant biofilm (lane 3), eDNA derived from Δ*dns*Δ*xds* mutant biofilm (lane 4), iDNA derived from Δ*dns* mutant biofilm (lane 5), eDNA derived from Δ*dns* mutant biofilm (lane 6), iDNA derived from Δ*xds* mutant biofilm (lane 7) and eDNA derived from Δ*xds* mutant biofilm (lane 8).

The extracellular nuclease activities of Xds and Dns and the altered biofilm formation in absence of both enzymes suggest that eDNA is a, so far uncharacterized, matrix component in *V. cholerae* biofilms. To test this hypothesis, we first investigated whether *V. cholerae* biofilms are sensitive to endo- and exonuclease treatment at an early and late time point. In case of the early time point, wild type and Δ*dns*Δ*xds* mutant were allowed to form biofilms for 21 h before the supernatant was removed and biofilms were incubated for an additional 3 h with DNase I, λExonuclease or a combination of both nucleases (Fig. 5A). Thus, the biofilms were allowed to form for a total time period of 24 h, which reflects the earliest time point with a significant difference in biofilm formation of the Δ*dns*Δ*xds* mutant compared with the wild type (Fig. 1B). Addition of DNase I or λExonuclease to *V. cholerae* cultures did not reduce growth or the viability of the cells as confirmed by measurements of the OD_600_ and cfu counts by plating (data not shown). Incubation with nuclease buffer alone was used as control condition reflecting biofilms without treatment. Nuclease sensitivity was determined by quantification of the residual biofilm after 3 h incubation by crystal violet staining (Fig. 5A). Treatment with DNase I or λExonuclease as well as with the combination of both enzymes significantly reduced the biofilm amount of the Δ*dns*Δ*xds* mutant to wild type levels and even lower. Interestingly, nuclease treatment of the wild type biofilm also decreased the biofilm amount with a significant twofold reduction in case of the combined treatment with both nucleases. These results demonstrate that wild type and the Δ*dns*Δ*xds* mutant biofilms are nuclease-sensitive at the early stages of biofilm development. We also investigated the nuclease sensitivity of the wild type and the Δ*dns*Δ*xds*, mutant at a late time point (72 h). However, at such late stages even a combined and prolonged treatment with DNase I and λExonuclease resulted in no significant decrease of the biofilm amount for all strains tested (Fig. S5). We speculate that biofilms at late stages are either too compact to allow externally added nuclease to be effective or other matrix components have accumulated and can compensate for the degradation of eDNA.

**Fig. 6:**
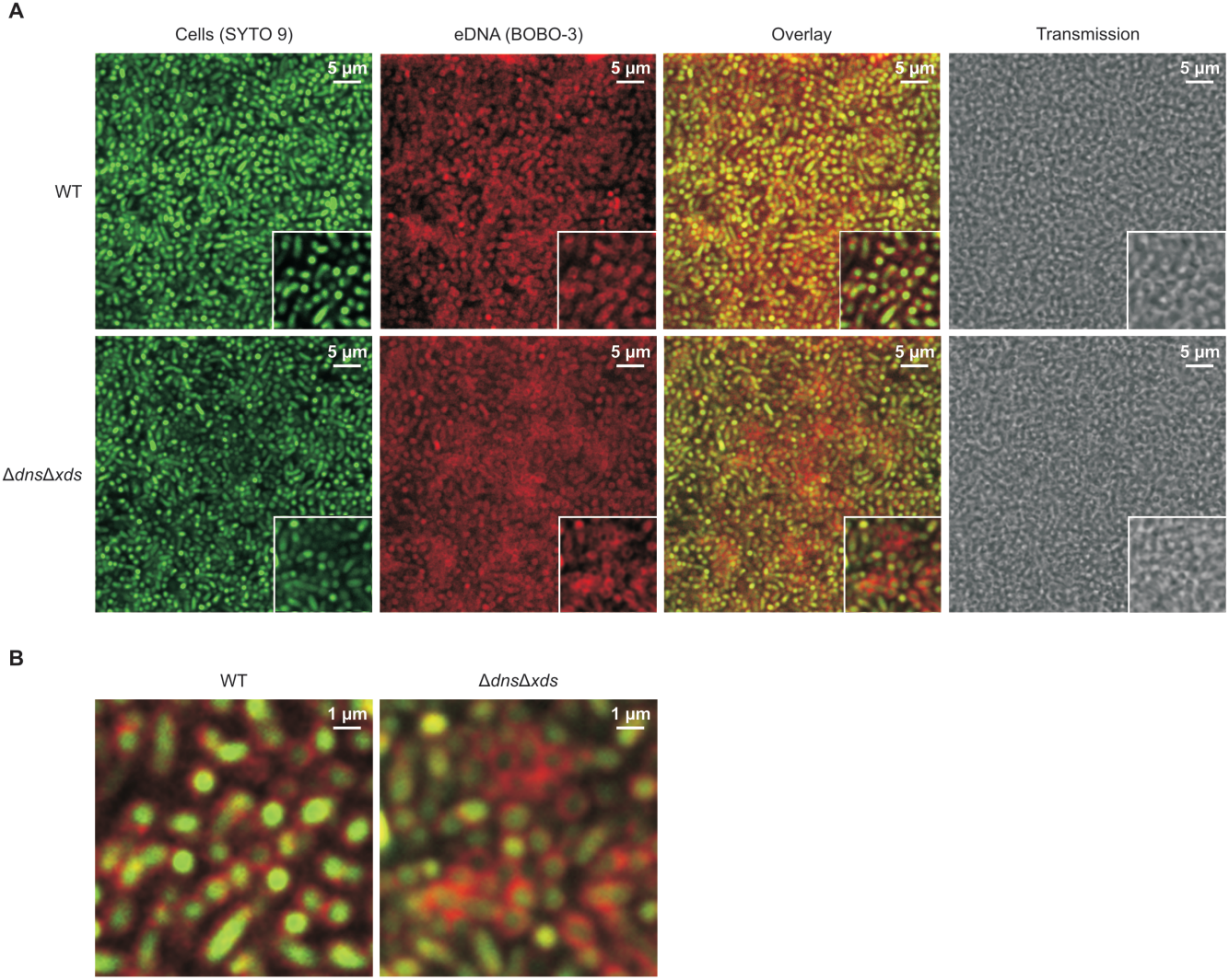
Visualization of eDNA in wild type and Δ*dns*Δ*xds* mutant biofilms. A. Shown are two-dimensional confocal laser scanning microscopy images of biofilms grown for 9 h in flow cell chambers and subsequently stained with SYTO 9 (green) and BOBO-3 (red). Bacterial cells appear in green and the eDNA in red. Representative zoomed images are shown in the bottom right corner. Images represent single optical sections acquired in comparable focal planes of the three-dimensional data sets. B. Shown are enlarged images of the overlay presented in panel (A) in the bottom right corner.

To visualize the eDNA, 9 h old flow cell biofilms were stained with SYTO 9 and BOBO-3 (Fig. 6). The membrane-permeant green fluorescent nucleic acid stain SYTO 9 generally labels all bacteria within the biofilm, while the red fluorescent nucleic acid dye BOBO-3 cannot penetrate through intact membranes and consequently only stains nucleic acids outside of the bacteria or membrane-compromised bacteria. Control experiments assessing the bacterial viability in the biofilm by live/dead staining using SYTO 9 and propidium iodide nucleic acid stains, the later of which only enters non-viable cells, indicate that only a few membrane-compromised bacteria are present at this time point (Fig. S6). As shown in Fig. 6, eDNA can be visualized in wild type and Δ*dns*Δ*xds* biofilms. In both cases, highest eDNA levels seem to be detected close to the cell surface decorating the bacterial cells. The eDNA in the wild type biofilm seemed to be more evenly distributed, whereas in the Δ*dns*Δ*xds* biofilm some hot spots with higher eDNA amounts can be detected. This indicates that Dns and Xds might be important for a homogenous distribution of the eDNA in biofilms, but makes it difficult to compare the overall amount of eDNA present. Therefore, statically grown 48 h old biofilms were dispersed, eDNA and intracellular DNA (iDNA) were separated from each other and the levels of eDNA and iDNA were quantified. At this time point the maximal difference in biofilm mass between wild type and Δ*dns*Δ*xds* was observed (Fig. 1D). The ratios of eDNA to iDNA for the wild type, Δ*dns*Δ*xds*, Δ*dns* and Δ*xds* mutant biofilms are shown in Fig. 5B. The ratios of the single mutants are not significantly altered compared with wild type. These results corroborate the hypothesis that deletion of one nuclease can be compensated by the remaining nuclease. The significant higher ratio in the Δ*dns*Δ*xds* mutant clearly indicates higher levels of eDNA in the biofilm of the double mutant compared with the wild type. Thus, the increased biofilm formation capacity of the Δ*dns*Δ*xds* mutant correlates with higher amounts of eDNA in the mutant biofilm.

Subsequently, the quality of iDNA and eDNA of the wild type, Δ*dns*Δ*xds*, Δ*dns* and Δ*xds* mutant biofilms was analysed on agarose gels (Fig. 5C). As expected, iDNA of all strains consisted of high molecular weight molecules migrating above 10 kb (Fig. 5C, lane 1, 3, 5 and 7). Extracellular DNA of the wild type was degraded to smaller fragments with the majority between 0.5 and 3 kb (Fig. 5C, lane 2). No high molecular weight eDNA was visible in the wild type sample. In contrast, the eDNA samples of the Δ*dns* and Δ*dns*Δ*xds* mutants contained a considerable amount of high molecular weight eDNA (Fig. 5C, lane 4 and 6). The Δ*xds* mutant sample revealed an intermediate phenotype with the intensity of high molecular weight DNA ranging between the wild type and the Δ*dns*Δ*xds* mutant (Fig. 5C, lane 8). A notable amount of smaller fragments was also visible in the eDNA samples of the mutants, especially for Δ*dns*Δ*xds* and Δ*xds*. The lack of smaller fragments in the Δ*dns* might be a result of the remaining Xds activity, which efficiently degrades these fragments down to the nucleotide level. As shown above, the supernatants of the Δ*dns*Δ*xds* mutant exhibit no detectable nuclease activity. Thus, the observed smaller-sized fragments most likely result from degradation of DNA by cytoplasmic nucleases probably originating from cell lysis. We doubt that nucleases released during the DNA preparation significantly contribute to the observed degradation, as dispersion of the biofilm had no detectable effect on cell viability confirmed via cfu measurements by plating and direct counts by microscopy (see *Experimental procedures*).

From these data, we speculate that the visible degradation of eDNA in the Δ*dns*Δ*xds* mutant originates from cytoplasmic nucleases as a consequence of spontaneous autolysis during biofilm growth, which could also be an important mechanism for eDNA release. Indeed, proteins that are involved in cell septation, cell wall recycling and autolysis have been recently linked to DNA release at early time points in *N. meningitidis* biofilms (Lappann *et al*., 2010). The authors observed the most prominent decrease of eDNA for mutants exhibiting reduced autolysis, i.e. an *ampD* mutant, encoding a N-acetylmuramyl-L-alanine amidase involved in cell wall recycling (Tipper, 1969; Huff *et al*., 1970; Gilpin *et al*., 1972; Singer *et al*., 1972; Oshida *et al*., 1995; Park, 1995; Firczuk and Bochtler, 2007; Vollmer *et al*., 2008). To test whether a deletion of *ampD* can also decrease eDNA levels in *V. cholerae* a Δ*dns*Δ*xds*Δ*ampD* mutant was constructed. The biofilm formation capacity of this triple mutant was analysed after 24 h using the static biofilm assay with crystal violet staining (Fig. 1B). The 24 h time point was chosen, as AmpD of *N. meningitidis* has been shown to impact eDNA release especially at early time points (Lappann *et al*.). Additionally, we observed growth defects of Δ*ampD* mutants in *V. cholerae* beyond 32 h, but not within 24 h (Fig. S1A). The triple mutant exhibited significantly reduced biofilm levels compared with the extracellular nuclease double mutant. A similar tendency, although not as pronounced, was also observed for a Δ*ampD* single mutant showing median biofilms levels lower than the wild type (Fig. S7). Expression of *ampD in trans* could partially restore biofilm production in the triple mutant resulting in a slight increase, but the levels of the Δ*dns*Δ*xds* mutant could not be reached (Fig. 1B). It has to be noted that expression of *ampD in trans* resulted in a slight growth defect that could also impact biofilm formation (Fig. S1A). Overall, these results strengthen the idea that at least one source of eDNA in bacterial biofilms is due to autolysis of cells.

### The two extracellular nucleases are essential for utilization of eDNA as phosphate source

**Fig. 7:**
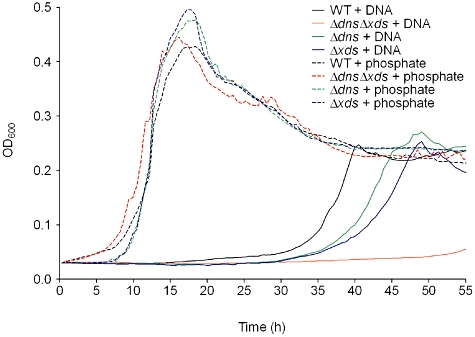
The two extracellular nucleases are important for utilization of DNA as phosphate source. Shown are the growth kinetics of wild type (black), Δ*dns*Δ*xds* (red), Δ*dns* (green) and Δ*xds* (blue) mutant monitored over 55 h in either M9 Tris glucose with inorganic phosphate (dashed lines) or M9 Tris glucose supplemented with herring sperm DNA (2.5 mg ml^−1^) as the sole source of phosphate (solid lines). Values represent medians from at least six independent measurements.

Besides the presence in the bacterial biofilm matrices, eDNA is also an abundant polymer in the aquatic ecosystem (Paul *et al*., 1987; Trevors, 1996; Dell'Anno and Danovaro, 2005). Therefore, eDNA could represent an important nutrient source for *V. cholerae* in this environment. For *P. aeruginosa* it has been recently shown that an extracellular nuclease is required for utilization of DNA as sole source for carbon, nitrogen and phosphate (Mulcahy *et al*., 2010). In that study the most pronounced growth phenotypes were observed using conditions with DNA as the sole source of phosphate. Thus, we determined whether *V. cholerae* can utilize DNA using Xds and Dns by growth assays under conditions of phosphate limitation. The growth of the wild type, Δ*dns*Δ*xds*, Δ*dns* and Δ*xds* mutants was monitored for 55 h in glucose minimal medium either containing inorganic phosphate and no DNA, or lacking inorganic phosphate but supplemented with herring sperm DNA (Fig. 7). In medium containing inorganic phosphate the wild type and all mutants showed similar growth abilities. Under conditions with DNA as the sole phosphate source growth was observed for the wild type after a long lag-phase. No growth was detected for the double mutant. Both single mutants were able to use DNA as the sole phosphate source, but exhibited a pronounced delay in growth compared with the wild type. Expression of *dns* and *xds in trans* using the IPTG-inducible vector pMMB could not be achieved in these experiments, as glucose served as a carbon source resulting in catabolite repression. We have tried to perform the growth assays under phosphate limiting conditions with different carbon sources (i.e. glycerol or maltose). Unfortunately, either no significant growth could be observed or precipitations occurred after 24 h, which tremendously affected the OD_600_ measurements. To overcome this limitation of the complementation analysis, we incubated DNA solutions with supernatants harbouring Dns or Xds activity, before their use as the sole source of phosphate in growth experiments with the double mutant (Fig. S1C). DNA incubated with a supernatant lacking Dns or Xds activity served as a control. In these growth experiments pre-incubation of the DNA with supernatants harbouring Dns or Xds activity allowed faster growth to higher optical densities of the double mutant compared with the control condition (Fig. S1C). The observable growth of the double mutant under control conditions is most likely due to phosphate added together with the DNA solution, which contained culture supernatants with 6.5 mM phosphate. Overall, these data are consistent with the results of growth kinetics obtained for the single mutants in Fig. 7. In summary, presence of each nuclease alone already allows *V. cholerae* to utilize DNA as a phosphate source, but presence of both activities is required to obtain the best growth under these conditions. Absence of both phosphate sources, inorganic phosphate and DNA, did not allow any growth for wild type and mutant strains (data not shown), indicating that phosphate was the limiting factor in all growth assays.

We also performed growth experiments using DNA as a sole source of carbon or nitrogen. However, even the wild type showed no visible growth using final DNA concentrations of up to 10 mg ml^−1^. We did not pursue these growth experiments, because addition of such high amounts of DNA resulted in a general growth defect in glucose minimal medium (Fig. S1B).

Based on these results, we investigated if *dns* and *xds* are expressed at higher levels under conditions of phosphate limitation. PhoA activities during planktonic growth were measured from wild type carrying the *dns-phoA* or *xds-phoA* transcriptional fusion grown in minimal medium containing high (65 mM) or low (6.5 mM) concentrations of inorganic phosphate (Fig. S8A). A 10-fold reduction of inorganic phosphate to 6.5 mM resulted in a severe growth delay of the wild type (Fig. S8B) and was the lowest phosphate concentration, which still allowed enough growth to acquire a substantial amount of cell density to measure PhoA activity. As shown in Fig. S8A a three- to five-fold higher activity for *dns-phoA* and *xds-phoA* was observed under conditions of phosphate limitation.

In summary, these results demonstrate that DNA can serve at least as a phosphate source allowing growth of *V. cholerae* under phosphate limiting conditions and that the two extracellular nucleases play a crucial role in this process. Accordingly, both nucleases are induced under phosphate limiting conditions.

### Impaired detachment from biofilms results in reduced colonization fitness of the Δ*dns*Δ*xds* mutant

**Fig. 8:**
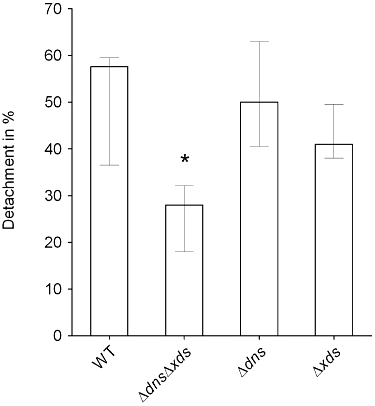
Deletion of *dns* and *xds* reduces the detachment in *V. cholerae* biofilms. Static biofilms were allowed to form for 40 h, subsequently the planktonic cell supernatant was replaced by cell free spent LB. The number of cells detached from the biofilm was determined after 3 h. The bars represent the percentage of detached cells to the overall cell number in the biofilm. Shown are the medians of at least nine independent measurements. The error bars indicate the interquartile range. The detachment rate of the Δ*dns*Δ*xds* mutant is significantly different compared with the wild type (*P < 0.05).

Based on the high amounts of compact biofilm produced by the Δ*dns*Δ*xds* mutant, we speculated that the mutant might be impaired for detachment from biofilms. To test whether this is true, wild type, Δ*dns*Δ*xds*, Δ*dns* and Δ*xds* mutants were allowed to form static biofilms for 40 h before the detachment rate within a 3 h time period was determined (Fig. 8). This time point was chosen, because the biofilm mass of the wild type increased steadily until 40 h in the static biofilm assay, but then remained almost at the same level (Fig. 1). The detachment rate in the Δ*dns*Δ*xds* mutant biofilm was significantly reduced compared with levels observed for the wild type. Although statistically not significant, a tendency towards decreased detachment was also observable for the *dns* and *xds* single mutants.

**Fig. 9:**
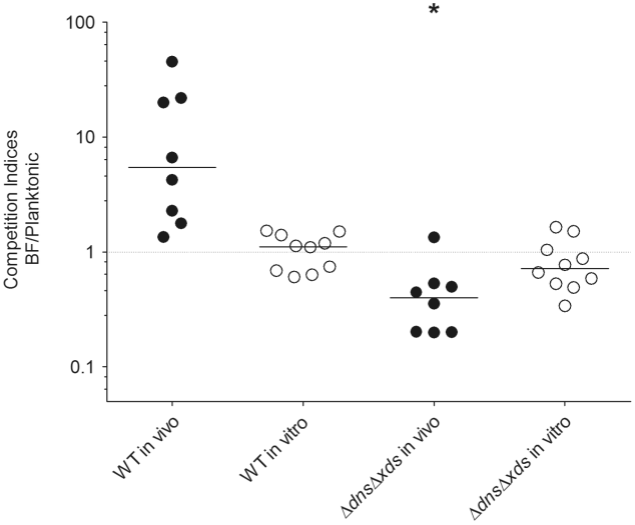
Extracellular nucleases are important for the colonization fitness of *V. cholerae* biofilms *in vivo*. Planktonic Δ*dns*Δ*xds* mutant cells out-compete their biofilm-derived counterparts (BF) in the small intestine of mice, whereas the opposite is true for wild type. In both tested cases the planktonic cells were *lacZ* negative and the biofilm-derived cells *lacZ* positive. Results are shown as the competitive indices (CI) *in vivo* using the infant mouse model (filled circles) and *in vitro* in LB shaking cultures (open circles). Each circle represents the CI from a single assay. Horizontal lines indicate medians for each data set. The *in vivo* CI of the Δ*dns*Δ*xds* mutant is significantly different compared with the wild type (*P < 0.05).

Biofilms are a likely form in which *V. cholerae* enters the human host, but after passage through the gastric barrier the bacteria have to detach from the biofilm to adhere and penetrate through the mucosal layer aided by motility (Freter and Jones, 1976; Freter and O'Brien, 1981; Huo *et al*., 1996; Colwell *et al*., 2003; Zhu and Mekalanos, 2003; Butler and Camilli, 2005). The low detachment rate of the Δ*dns*Δ*xds* mutant *in vitro* could also affect the dissolution of biofilm clumps *in vivo* resulting in reduced colonization fitness. Previous reports already demonstrated that inactivation of one or both extracellular nucleases do not reduce virulence of planktonic *V. cholerae* (Focareta and Manning, 1991a). To test whether the colonization fitness changes by using biofilms as inoculum, we performed competition experiments comparing the colonization efficiency with differentially marked biofilm clumps and planktonic cells of either the wild type or the Δ*dns*Δ*xds* mutant. Presence of uniform pieces of biofilm clumps in the inoculum for *in vivo* experiments, with approximately 50 to 100 cells for wild type and double mutant, was confirmed by microscopy (Fig. S9). The original output cfu of the planktonic and biofilm derived cells of the wild type and *ΔdnsΔxds* mutant obtained in these experiments are provided separately in Fig. S10. Based on the results, the planktonic cells of the wild type and the Δ*dns*Δ*xds* mutant exhibit a comparable colonization fitness. Thus, consistent with the data from Focareta *et al*. a direct colonization defect of planktonic cells of the Δ*dns*Δ*xds* mutant can be excluded [(Focareta and Manning, 1991a) and Fig. S10]. The competition indices resembling the ratio of biofilm derived to planktonic cells of the wild type or the Δ*dns*Δ*xds* mutant are shown in Fig. 9. Wild type biofilms out-competed wild-type planktonic cells about fivefold in the colonization assay, whereas wild type biofilms and planktonic cells showed comparable growth *in vitro*. This result is consistent with a previous report demonstrating that growth in biofilms induces a hyperinfectious phenotype in *V. cholerae* (Tamayo *et al*., 2010). In contrast, when inocula composed of the Δ*dns*Δ*xds* mutant were used, the biofilms showed a reduced colonization compared with planktonic cells. As is evident from the results shown in Fig. 8, the Δ*dns*Δ*xds* mutant detaches at low rates from its biofilms. Thus, the observed colonization defect of Δ*dns*Δ*xds* mutant biofilms is most likely due to inefficient dissolution of biofilm clumps within the lumen of the small intestine.

## Discussion

The facultative pathogen *V. cholerae* transits between the pathogenic lifestyle in the gastrointestinal tract of the human host and the persistence as a natural inhabitant of aquatic ecosystems. Thereby the aquatic environment serves as an environmental reservoir for *V. cholerae* between the seasonal outbreaks (Faruque *et al*., 1998; Alam *et al*., 2007). Within aquatic ecosystems biofilms on chitinous surfaces are likely to be a preferred survival mode of *V. cholerae*. The biofilm formation capacity of *V. cholerae* is well documented and numerous studies have investigated structural factors including flagella, pili and exopolysaccharide synthesis, as well as the regulatory processes that control their expression.

In this study we demonstrated that the activity of the two extracellular deoxyribonucleases Dns and Xds is required for the development of a normal three-dimensional architecture of the biofilm. Absence of the nucleases causes a massive and unco-ordinated accumulation of the biofilm mass finally resulting in compact, thick and unstructured biofilms. Dns and Xds are solely responsible for the extracellular nuclease activity of *V. cholerae*, as no detectable extracellular nuclease activity was observed for the Δ*dns*Δ*xds* mutant in any assay described in this study. Detection of both activities in the supernatants also indicates that both nucleases are secreted rather than surface associated, which had previously only been reported for Dns (Focareta and Manning, 1987; Blokesch and Schoolnik, 2008). Although single mutants of the extracellular nucleases already showed increased biofilm amounts, and slightly reduced detachment rates compared with the wild type, the most prominent effects throughout the study were observed when both nucleases were deleted. These data show that Dns and Xds can at least partially compensate for each other and may act somewhat synergistically.

This study revealed two distinct differences between the nucleases regarding their enzymatic activity and transcriptional regulation. In order to discriminate the enzymatic activities we analysed the degradation of circular or linearized DNA added to culture supernatants containing either Dns or Xds. Dns was able to degrade both types of DNA and can therefore be classified as an endonuclease as previously suggested (Altermark *et al*., 2007; Blokesch and Schoolnik, 2008; Niiranen *et al*., 2008). In contrast, Xds exhibits an exonuclease activity, as it only targeted linearized DNA. Based on the observed temporal degradation pattern of linearized DNA the overall DNase activity of Dns seemed to be higher compared with Xds. This is consistent with previous reports that Dns hinders natural transformation by degradation of free eDNA, while Xds only contributes marginally to this effect (Blokesch and Schoolnik, 2008).

Consistent with a previous report we confirmed that expression of *dns* is regulated via HapR acting as a repressor (Blokesch and Schoolnik, 2008). In contrast, our results also demonstrate that *xds* transcription is independent of HapR. Consequently, *dns* is co-regulated with *vps* genes (Hammer and Bassler, 2003; Blokesch and Schoolnik, 2008). Furthermore, the temporal expression of *dns* and *xds* along the biofilm development was investigated and revealed different expression patterns for the two nucleases. The expression of *dns* was relatively high at the early and late stages of the biofilm, with a significant decrease in between. In contrast, *xds* expression increased steadily with the highest levels at the late time point. The expression patterns in combination with the other results of this study suggest that under low cell density, for example during initial stages of biofilm formation, highly expressed *vps* genes drive the production of VPS and Dns degrades available eDNA into smaller sized fragments. Xds may participate in the degradation of eDNA at this stage of biofilm formation, but our results imply that Dns is the dominant nuclease showing very efficient DNA degradation. This could also explain that throughout the study the deletion of *dns* had slightly more pronounced effects on biofilm formation and morphology especially at earlier time points compared with the deletion of *xds*. As the biofilm matures, accumulation of autoinducers and increase of cell density then results in repression of *vps* and *dns* transcription, while *xds* remains expressed. Thus, at these stages of the biofilm Xds might become the dominant nuclease resulting in further degradation of eDNA down to the nucleotide level. At the latest time point high expression of both nucleases was observed, indicating that the nucleases are induced in late stages of mature biofilms. One signal for induction of both nucleases at these late stages might be nutrient limitation. As demonstrated in this study, both nucleases are induced under conditions of phosphate limitation. Because both nucleases are also involved in the detachment process, the induction of both nucleases upon phosphate limitation could also trigger the dissolution of biofilms. Degradation of eDNA might not only yield nucleotides as a source of phosphate, but could also generate free nucleosides. Indeed, *Vibrio* encodes genes involved in nucleoside catabolism, which are controlled by the repressor CytR. Interestingly, in *V. cholerae* CytR represses also biofilm development and exopolysaccharide synthesis (Haugo and Watnick, 2002). Thus, genes encoding for nucleoside catabolism might be derepressed during biofilm formation in the aquatic environment and nucleosides could serve as a nutrient source for *V. cholerae* under these nutrient-limiting conditions.

Besides VPS, no other constituent of the *V. cholerae* biofilm matrix has been described so far. Based on the observations that the Δ*dns*Δ*xds* mutant shows increased biofilm formation without changing VPS production and that biofilms of *V. cholerae* are sensitive to nuclease treatment, we hypothesized that eDNA is an uncharacterized component of the *V. cholerae* biofilm matrix. We confirm the presence of eDNA by its visualization in biofilms using fluorescence microscopy and its isolation from the biofilm matrix. The enhanced biofilm production of the Δ*dns*Δ*xds* mutant compared with the wild type is likely due to the elevated levels of eDNA found in the extracellular nuclease mutant biofilm matrix. Such increased eDNA levels might allow enhanced attachment and recruit more cells to the biofilm. Another explanation could be that bacteria as well as VPS are trapped and retain in the biofilm. The correlation between eDNA and biofilm levels might also explain the observed increase in biofilm production upon presence of a plasmid. Because plasmid DNA could also contribute to eDNA in the biofilm, the presence of a plasmid could result in more eDNA and consequently more biofilm. The mechanisms of the eDNA release are still poorly understood, but autolysis is hypothesized to be a major source. In *E. faecalis* two extracellular proteases and in *Staphylococcus aureus* the *cid*/ *lrg* operons are involved in an autolysis-dependent eDNA release (Thomas *et al*., 2008; Mann *et al*., 2009). In *N. meningitidis*, where eDNA is important for the initiation of biofilm formation, *ampD* mutants with reduced autolysis also exhibit significantly diminished biofilm formation (Lappann *et al*., 2010). Consistent with this report, deletion of *ampD* also reduced biofilm formation in *V. cholerae*, indicating that autolysis is at least one origin of eDNA. A second source might be outer membrane vesicles (OMVs), which are naturally secreted by a variety of Gram-negative bacteria including *V. cholerae* (Chatterjee and Das, 1967; Kondo *et al*., 1993; Schild *et al*., 2008b). DNA within OMVs was previously observed for *N. gonorrhoeae*, *P. aeruginosa* and *E. coli* (Dorward *et al*., 1989; Yaron *et al*., 2000; Renelli *et al*., 2004). Future studies have to investigate whether OMVs derived from *V. cholerae* also contain DNA and are secreted during biofilm formation.

Extracellular DNA has been recently found in biofilms of other bacteria and shown to perform different roles in the biofilm development of the respective microorganisms. By analysing not only eDNA, but also its degradative enzymes Dns and Xds, we were able to characterize multiple roles for eDNA in the *V. cholerae* biofilm. We found that eDNA and the extracellular nucleases Dns and Xds are essential for the development of the normal three-dimensional biofilm structure with holes, pillars and fluid-filled channels. Thus, eDNA in *V. cholerae* not only facilitates initial attachment as described for *Pseudomonas* and *Neisseria*, but is also an important structural factor in biofilms.

Extracellular DNA is not only a ubiquitous component of the soil, but also of marine and freshwater habitats where it can reach concentrations of 88 µg l^−1^ (Vlassov *et al*., 2007). In addition, DNA is also a significant component of the small-intestinal mucus (Ferencz *et al*., 1980). Because *V. cholerae* transits between both environments along its life cycle, it might have evolved systems to utilize eDNA as a carbon, nitrogen and phosphate source. Acquisition of nutrients is crucial for persistence in the nutrient-poor aquatic ecosystem. Thus, *V. cholerae* can for example utilize chitin as a carbon and nitrogen source (Meibom *et al*., 2004). However, free phosphate is an especially limiting factor for survival of most organisms including *V. cholerae* in the aquatic reservoirs (Pratt *et al*., 2009). A recent study revealed that inorganic phosphate declines 1600-fold from 160 ppm in cholera patient stool to 0.1 ppm in pond water (Nelson *et al*., 2008). Hence, *V. cholerae* faces a severe drop in free inorganic phosphate during the transition from the host into the aquatic environment. Thus, acquisition of phosphate is crucial upon entry into the aquatic lifestyle. As demonstrated in this study, *V. cholerae* is capable of utilizing DNA as a phosphate source due to the activity of the extracellular nucleases Dns and Xds. Recently, *xds* was identified as a late *in vivo* induced gene (Schild *et al*., 2007). Strikingly, a number of such genes induced late during infection have been demonstrated to increase fitness of *V. cholerae* after release into the environment. As the formation of biofilms is thought to play a key role in environmental survival, Xds and Dns could be important for nutrient acquisition in the host as well as in the aquatic environment due to degradation of DNA.

Extracellular DNA represents also a dynamic gene pool from which bacteria can obtain genetic information by horizontal gene transfer (Vlassov *et al*., 2007). The mosaic-structured genome of *V. cholerae* implicates the importance of horizontal gene transfer during evolution (Heidelberg *et al*., 2000). Virulence of *V. cholerae* is most likely the result of a series of horizontal gene transfer events that allowed a benign marine bacterium to evolve into a human pathogen (Reidl and Klose, 2002). The newly emerged serogroup O139 has evolved from an O1 El Tor ancestor through the acquisition of the genes encoding for the O139 antigen and capsule (Bik *et al*., 1995). Interestingly, *V. cholerae* induces natural competence while growing on chitin (Meibom *et al*., 2005). This implicates the possibility of horizontal gene transfer while *V. cholerae* is forming biofilms on natural chitinous material in the aquatic environment. A successful serogroup conversion of an O1 recipient by an O139 donor and transfer of classical biotype cholera toxin genes from *V. cholerae* serotype O141 to O1 biotype El Tor were recently demonstrated by co-culturing both strains on chitin surfaces (Blokesch and Schoolnik, 2007; Udden *et al*., 2008). The presence of eDNA in *V. cholerae* biofilms, as demonstrated in this study, indicates that *V. cholerae*, like other bacteria, contributes to the pool of extracellular genetic material available for different strains and species and strengthens the current model of the O1-to-O139 conversion and acquisition of virulence factors in the evolution of *V. cholerae*.

*Vibrio cholerae* biofilm clumps formed in the aquatic reservoir are an important source for new cholera outbreaks (Huo *et al*., 1996; Hall-Stoodley and Stoodley, 2005; Pruzzo *et al*., 2008). This is highlighted by the observation that filtration of water reducing the concentration of particles greater than 20 µm in size can reduce cholera incidence rates in endemic areas by 48% (Colwell *et al*., 2003). Biofilm-associated *V. cholerae* are better protected against acids or bile salts and biofilms most likely allow a higher number of bacteria to reach the small intestine (Nalin *et al*., 1978; 1979; Zhu and Mekalanos, 2003; Hartley *et al*., 2006). However, at the primary site of colonization *V. cholerae* has to detach from the biofilm and use flagellar motility to attach and penetrate through the mucosal layer as well as to induce full virulence (Freter and Jones, 1976; Freter *et al*., 1981; Butler and Camilli, 2004; 2005; Lauriano *et al*., 2004; Liu *et al*., 2008; Moisi *et al*., 2009). It has been previously demonstrated that the unregulated VPS production of *hapR* mutants results in lower detachment rates, which correlates with a reduced colonization fitness of *hapR* mutant biofilms (Zhu and Mekalanos, 2003; Liu *et al*., 2007). As shown in this study, extracellular nucleases contribute to an efficient detachment from the mature biofilm. Consistently, a colonization defect for biofilms of the Δ*dns*Δ*xds* mutant compared with planktonic cells was observed. In contrast, wild type biofilms out-competed planktonic cells, which is consistent with a recent report demonstrating that growth in a biofilm induces a hyperinfectious phenotype in *V. cholerae* (Tamayo *et al*., 2010). Because the biofilms of the mutant and wild type were dispersed into clumps of similar size with approximately 50–100 cells, we doubt that the thick and compact structure of the mutant biofilm by itself hinders the bacteria to efficiently colonize the small intestine. Along with the observed nuclease sensitivity of biofilms and the results of the detachment assay, we rather speculate that Dns and Xds are involved in the dissolution step of biofilm-derived cells *in vivo* via degradation of eDNA. To our knowledge, this is the first report of enzymes that are directly involved in the detachment process by degrading material of the *V. cholerae* biofilm matrix.

In summary, we identified eDNA as an important, versatile matrix component of *V. cholerae* biofilms. Extracellular DNA, together with the corresponding modulatory extracellular nucleases Dns and Xds, are involved in several processes of *V. cholerae* during life in a biofilm, including the development of a typical biofilm architecture, detachment from biofilms, nutrient acquisition and the *in vivo* colonization fitness of biofilm clumps after ingestion by the host.

## Experimental procedures

### Ethics statement

This study was carried out in strict accordance with the recommendations in the Guide for the Care and Use of Laboratory Animals of the ‘Bundesgesetzblatt fuer die Republik Oesterreich’ and the National Institutes of Health. The protocol was approved by the Committee on the Ethics of Animal Experiments of the University of Graz as well as the Austrian Federal Ministry for Science and Research BM.W-F (Permit Number: 39/158 ex2000/10). Intragastric infections were performed under isoflurane anaesthesia and all efforts were made to minimize suffering.

### Bacterial strains and culture conditions

Bacterial strains and plasmids used in this study are listed in Table 1. The clinical isolate *V. cholerae* O1 El Tor C6709 was used as wild type (Roberts *et al*., 1992). *E. coli* DH5αλ*pir* was used for maintenance of plasmids and SM10λ*pir* to deliver plasmids to *V. cholerae* by conjugation (Kolter *et al*., 1978; Hanahan, 1983; Miller and Mekalanos, 1988). Unless stated otherwise strains were grown in LB broth with aeration at 37°C or for biofilm experiments under static conditions at room temperature (RT). Antibiotics and other supplements were used in the following final concentrations: streptomycin (Sm), 100 µg ml^−1^; ampicillin (Ap), 100 µg ml^−1^ or 50 µg ml^−1^ in combination with other antibiotics; kanamycin (Km), 50 µg ml^−1^; isopropyl-β-thiogalactopyranoside (IPTG), 0.5 mM; glucose (Gluc), 0.2%; sucrose (Suc), 10%, 30 µg ml^−1^ 5-bromo-4-chloro-3-indolyl-β-d-galactopyranoside (Xgal).

**Table 1:** Strains used in this study

Strain/plasmid	Description	Reference
*E. coli*		
DH5αλ*pir*	F^-^Φ80*ΔlacZ*Δ*M15*Δ(*argF lac*)*U169 deoR recA1 endA1 hsdR17* (r_K_^-^m_K_^+^) *supE44 thi-1 gyrA69 relA1, λpir*R6K, Ap^r^	Hanahan (1983)
SM10λ*pir*	*thi thr leu tonA lacY supE recA*::RPA-2-Te::Mu *λpir*R6K, Km^r^	Miller and Mekalanos (1988)
*V. cholerae*		
C6709	WT, O1 El Tor Inaba, clinical isolate, 1991 Peru, *tcpA*+*ctx*+*hapR*+, spontaneous Sm^r^	Roberts *et al*. (1992)
C6709*lacZ*	C6709, *lacZ*::res-*neo-sacB*-res	Tamayo *et al*. (2008)
Δ*dns*	Deletion of *dns* in C6709, Sm^r^	This study
Δ*hapR*	Deletion of *hapR* in C6709, Sm^r^	This study
Δ*vpsA*	Deletion of *vpsA* in C6709, Sm^r^	This study
Δ*xds*	Deletion of *xds* in C6709, Sm^r^	This study
Δ*dns*Δ*xds*	Deletion of *xds* in Δ*dns*, Sm^r^	This study
Δ*dns*Δ*xds*Δ*ampD*	Deletion of in *ampD* in Δ*dns*Δ*xds*, Sm^r^	This study
Δ*dns*Δ*xds lacZ*	Δ*dns*Δ*xds, lacZ*::res-*neo-sacB*-res	This study
C6709*dns*::pGPphoA	Insertion of pGPphoAdns in *dns* of C6709, Sm^r^, Ap^r^	This study
Δ*hapRdns*::pGPphoA	Insertion of pGPphoAdns in *dns* of Δ*hapR*, Sm^r^, Ap^r^	This study
C6709*vpsA*::pGPphoA	Insertion of pGPphoAvpsA in *vpsA* of C6709 Sm^r^, Ap^r^	This study
Δ*hapRvpsA*::pGPphoA	Insertion of pGPphoAvpsA in *vpsA* of Δ*hapR*, Sm^r^, Ap^r^	This study
C6709*xds*::pGPphoA	Insertion of pGPphoAxds in *xds* of C6709, Sm^r^, Ap^r^	This study
Δ*hapRxds*::pGPphoA	Insertion of pGPphoAxds in *xds* of Δ*hapR*, Sm^r^, Ap^r^	This study
Plasmids		
pCVD442	*oriR6K mobRP4 sacB*, Ap^r^	Donnenberg and Kaper (1991)
pGPphoA	pGP704 with promotorless *phoA* of SM10λ*pir*, Ap^r^	Moisi *et al*. (2009)
pMMB	pMMB67EH, IncQ broad-host-range low-copy-number cloning vector, IPTG inducible, Ap^r^	Morales *et al*. (1991)
pBAD24	araBADp cloning vector, Ap^r^	Guzman *et al*. (1995)
pCVD442ΔampD	pCVD442::Δ*ampD*, Ap^r^	This study
pCVD442Δdns	pCVD442::Δ*dns*, Ap^r^	This study
pCVD442ΔhapR	pCVD442::Δ*hapR*, Ap^r^	This study
pCVD442ΔvpsA	pCVD442::Δ*vpsA*, Ap^r^	This study
pCVD442Δxds	pCVD442::Δ*xds*, Ap^r^	This study
pGPphoAdns	pGPphoA with ‘*dns*’ fragment of C6709, Ap^r^	This study
pGPphoAvpsA	pGPphoA with ‘*vpsA*’ fragment of C6709, Ap^r^	This study
pGPphoAxds	pGPphoA with ‘*xds*’ fragment of C6709, Ap^r^	This study
pampD	*ampD* of C6709 in pMMB, Ap^r^	This study
pdns	*dns* of C6709 in pMMB, Ap^r^	This study
pxds	*xds* of C6709 in pMMB, Ap^r^	This study

### Construction of suicide plasmids, deletion mutants and expression plasmids

Chromosomal DNA was isolated using the method described by Grimberg *et al*. (Grimberg *et al*., 1989). For phenol extraction phase lock gel tubes (Eppendorf) were used and the chromosomal DNA was obtained by ethanol and salt precipitation. Plasmids, digested plasmids and PCR products were purified using the QIAquick PCR purification, the QIAquick gel extraction, or the QIAprep Spin Mini Kit (Qiagen). PCRs for subcloning were performed with Phusion polymerase (New England Biolabs), for all other reactions *Taq* DNA polymerase (New England Biolabs) was used. Oligonucleotides used in this study are listed in Table 2.

**Table 2:** Oligonucleotides used in this study

Oligonucleotides	Sequence (5′ to 3′)
VC0470_XbaI_1	CGTCTAGATAGCCAAGATCGCCGAAA^a^
VC0470_2	CGTCTTTTATGTGATGGGCAGAATCTCACC
VC0470_3	CTGCCCATCACATAAAAGACGTAGATAAGTAGGTTTTT
VC0470_XbaI_4	CGTCTAGAGGTATGGCTGATCGTTGTGA
VC0583_SacI_1	TTTGAGCTCTGCGCGTAGTCGATACCG
VC0583_EcoRI_2	TTTGAATTCCATAGGGGTATATCCTTGCCA
VC0583_EcoRI_3	TTTGAATTCTAGTTTCTTGGGCAGCACAAA
VC0583_XbaI_4	TTTTCTAGATACGCGTCATACCGGAAA
VC0917_XbaI_1	TTTTCTAGATTATTTTACGCGATAAGA
VC0917_EcoRI_2	TTTGAATTCCACTTCCCCACATCCTCTT
VC0917_EcoRI_3	TTTGAATTCTAGACTCATCAGGGGATGACA
VC0917_SacI_4	TTTGAGCTCACACGAGGATGGCGGTT
VC2621_SacI_1	TTAGAGCTCGAATACCAACTGCATTTCAT
VC2621_SphI_2	TTTGCATGCCATGATGTACCTTCTCCTCCCT
VC2621_SphI_3	TTTGCATGCTAGAAAGTGGCATTTTTGATA
VC2621_XbaI_4	TTATCTAGAAGAGCTTGGCAAAATGGGCA
ampD_SacI_1	TTTGAGCTCGAGCAATCGGCACTGG
ampD_EcoRI_2	AAAGAATTCCATCCTTACTCCTTGCTTATA
ampD_EcoRI_3	AATGAATTCTAGGTTTTAGTTGGTGCGAAA
ampD_XbaI_4	AATTCTAGATGAAGATTGGTCTATTTTATG
dns_SacI	TTTGAGCTCACCTTTGCCGCCCCC
dns_KpnI	TTTGGTACCTCAGTTCGGGCATTGCTCACG
vpsA_SacI	AAAGAGCTCTGTCAAGACAATCGTTTT
vpsA_KpnI	TTTGGTACCGACTAATTTCACCGT
xds_SacI	TTTGAGCTCCGCGATAAAGTGGTGAAGCTG
xds_KpnI	TTTGGTACCTTTCTAGCGACGGCGACG
VC0470_SacI_5′	TTTGAGCTCCTACTTATCTACGTCTTTTTAG
VC0470_XbaI_3′	AAATCTAGATTCTGCCCATCAGTTCGG
VC2621_SacI_5′	TTTGAGCTCAAATAGGGAGGAGAAGGTAC
VC2621_XbaI_3′	TTTTCTAGACTTTCTAGCGACGGCGACG
ampD_SacI_5′	AAAGAGCTCTATAAGCAAGGAGTAAGGATG
ampD_XbaI_3′	AAATCTAGAGGGAACTCAACCAAGC

a Restriction sites are underlined.

Constructions of in-frame deletion mutants were carried out as described by Donnenberg and Kaper (Donnenberg and Kaper, 1991). PCR fragments of approximately 800 bp upstream and downstream of the gene of interest were PCR-amplified using the oligonucleotide pairs A_B_1 and A_B_2 or A_B_3 and A_B_4, in which A stands for the gene and B for the restriction site used. In-frame deletion of *dns* was obtained by splicing overlap extension PCR (Horton *et al*., 1989) using oligonucleotide pairs VC0470_XbaI_1 and VC0470_2 as well as VC0470_3 and VC0470_XbaI_4. After digestion of the PCR fragments with the appropriate restriction enzyme (New England Biolabs) indicated by the name of the oligonucleotide, they were ligated into the SacI/XbaI-digested pCVD442 or XbaI-digested pCVD442 respectively.

Derivatives of pGPphoA were constructed to obtain chromosomal transcriptional fusions of *phoA* to respective gene transcripts. Gene fragments of *dns*, *xds* and *vpsA*, containing the translational stop codon of the respective gene, were amplified by PCR using oligonucleotide pairs dns_SacI and dns_KpnI, xds_SacI and xds_KpnI or vpsA_SacI and vpsA_KpnI. PCR products were digested with SacI and KpnI and ligated into the pGPphoA vector that had been digested with the same enzymes.

For the expression plasmids the respective gene was PCR-amplified using the oligonucleotide pairs designated in the way A_B_5′ and A_B_3′, in which A stands for the gene and B for the restriction site used. The PCR fragment was digested with SacI and XbaI, and ligated in the similar digested pMMB expression vector. After transformation of the ligation products in DH5αλ*pir*, Ap^r^ colonies were characterized by PCR and/ or restriction analysis (data not shown).

In case of the suicide plasmid derivatives the correct constructs were transformed into SM10λ*pir* and mobilized into *V. cholerae* by conjugation, which was achieved by cross-streaking donor and recipient on LB agar plates followed by incubation for 6 h on 37°C. *V. cholerae* conjugants were purified via selection for Sm^r^ and Ap^r^ colonies. In the case of pCVD442 derivatives sucrose selection was used to obtain Ap^s^ colonies. Chromosomal insertions or deletions were confirmed by PCR (data not shown).

### Static biofilm assay with crystal violet staining

Static biofilm assays were performed as previously published (Watnick and Kolter, 1999) with following modifications. An overnight culture of the respective strain was diluted 1:150 in LB broth. One hundred and fifty microlitres of this dilution was placed into sterile polystyrene 96 well U bottom microtiter plate wells (Sterilin) and biofilm was grown for a time period of 12, 24, 40, 48 or 72 h at RT. Wells were subsequently rinsed six times with 200 µl dH_2_O with a mircoplate washer (LP41) and adhered bacteria were stained with 180 µl crystal violet solution (0.1%) for 10 min. The wells were again washed four times and the crystal violet stained biofilm was solubilized in 250 µl ethanol (96%). Wells loaded with LB broth only were included in every experiment as a negative control and showed no detectable crystal violet staining. Biofilm formation was then quantified by measuring an OD_595_ using a microplate reader (Biorad).

### Alkaline phosphatase (PhoA) assay

To determine the enzymatic activities for transcriptional *phoA* fusions, alkaline phosphatase assays were performed of the respective strains as described previously using cultures grown to an OD_600_ of ∼0.2 or ∼1, overnight cultures or cells derived from statically grown biofilms at the indicated time points (Schild *et al*., 2005; 2009). The activities are expressed in Miller Units, calculated as following: (A_405_ × 1000)/(A_600_ × ml × min).

### Flow cell biofilm experiments

For visualization of biofilms they were formed in three-channel flow cells with a modified version of the biofilm setup described by Sternberg *et al*. (Sternberg *et al*., 1999; Sternberg and Tolker-Nielsen, 2006). A coverslip 24 × 50 mm (Menzel-Glaeser) was used as substratum for biofilm growth. The respective overnight cultures were adjusted to OD_600_ = 0.1 using either LB or 50-fold diluted LB (2%) as described by Yildiz *et al*. (2004). Approximately 250 µl of the dilutions was inoculated per channel. After static incubation for 2 h at RT, flow of LB or 2% LB was initiated at a constant rate of 3 ml h^−1^ with the use of a Watson Marlow 205S. Biofilm was allowed to form at RT for a time period of 9 h using LB or 24 h using 2% LB. Images of attached bacteria or biofilms were recorded by confocal laser scanning microscopy.

### Fluorescent staining

To show biofilm morphology, biofilms were grown in flow cells and stained with SYTO 9. SYTO 9 from the Live/Dead BacLight Bacterial Viability kit (Invitrogen) was freshly diluted 400-fold in LB broth and approximately 250 µl of the dilution was injected into the flow cell channel. Biofilm was stained at RT for 20 min and images were recorded by confocal laser scanning microscopy.

For visualization of eDNA, flow cell biofilms were stained with a mixture of SYTO 9 and BOBO-3 (Invitrogen). SYTO 9 allows visualization of cells, whereas BOBO-3 is membrane impermeable and therefore specifically stains eDNA (Schleheck *et al*., 2009). 0.4 µl of each fluorescent dye was added to 1500 µl LB and approximately 250 µl of this mix was injected into the flow cell channel. Biofilm was stained at RT for 30 min and images were recorded by confocal laser scanning microscopy.

### Confocal laser scanning microscopy

Microscopy was performed using a Leica SP5 confocal microscope (Leica Microsystems, Mannheim, Germany) with spectral detection and a Leica HCX PL APO CS 40 × oil immersion objective (NA 1.25). Optical sectioning was performed in 0.2 µm steps.

SYTO 9 (Invitrogen) was excited at 488 nm and fluorescence emission was detected between 500–530 nm. Propidium Iodide (Invitrogen) was also excited with the 488 nm argon laser line and fluorescence emission was detected between 570–630 nm. BOBO-3 (Invitrogen) was excited at 561 nm and fluorescence emission was detected between 570–620 nm. Fluorescence signal of double labelled specimens and transmission images were acquired simultaneously. Images were recorded without differential interference contrast (DIC) optics. For visualization and processing of three-dimensional image data the Leica LAF and IMARIS software (volume rendering with shadow projection) was used. Quantitative analysis of the image stacks was performed using computer program COMSTAT (http://www.comstat.dk) (Heydorn *et al*., 2000; M. Vorregaard *et al*., pers. comm.). At least six image stacks from three independent experiments were used for the analysis.

### DNase activity assays

A DNase activity assay using plasmid or chromosomal DNA as substrate was performed essentially as described previously (Blokesch and Schoolnik, 2008; Mulcahy *et al*., 2010). Cells from overnight cultures of the respective *V. cholerae* strains were pelleted by centrifugation at 5000 *g* for 10 min and the supernatants were withdrawn. Twenty microlitres of the filter-sterilized supernatant was incubated at RT with 1 µg of purified circular plasmid DNA (pBAD24), linearized plasmid DNA (pBAD24 digested with EcoRI) or chromosomal DNA derived from *V. cholerae* wild type. Finally, samples were visualized on agarose gels (0.8%).

### Nuclease treatment of biofilms

Static biofilms were cultivated as described above. At the respective time points the wells were rinsed four times with spent LB (obtained from a 24 or 72 h old biofilm culture of the respective strain) before DNase I (AppliChem), λExonuclease (New England Biolabs) or in combination were added to a final concentration of 133 Kunitz units per ml. Addition of spent LB with nuclease buffer (50 mM Tris/HCl pH 7.5, 10 mM MgCl_2_, 50 µg µl^−1^ BSA, 5 mM CaCl_2_) served as a control. The biofilm was incubated with the respective solution for 3 or 7 h at RT respectively. Afterwards the wells were rinsed four times with distilled water and the remaining biofilm was stained with crystal violet and quantified as described above.

### eDNA and iDNA quantification

The eDNA and iDNA assay was performed essentially as previously published (Steinberger and Holden, 2005; Nakamura *et al*., 2008; Kreth *et al*., 2009; Harmsen *et al*., 2010). Briefly, an overnight culture of the respective strain was diluted 1:150 in LB broth. A borosilicate glass tube was inoculated with 1 ml of the resulting dilution and biofilm was allowed to form for 48 h under static conditions. Afterwards the biofilm was dispersed by treatment in an ultrasonic water bath for 1 min. Vortexing and treatment in the ultrasonic water bath was confirmed not to lyse cells or affect cell viability by several means including cfu measurements by plating and direct counts by microscopy. These controls have been conducted as described previously (Tamayo *et al*., 2010). Cells were removed by centrifugation for 10 min at 5000 *g*. The cell pellet was used for isolation of iDNA performing a standard chromosomal DNA preparation as described above. For the eDNA preparation the supernatant was transferred to a phase lock gel tube (Eppendorf) and DNA was isolated by phenol extraction followed by ethanol precipitation. Precipitated iDNA and eDNA pellets were suspended in 100 µl ddH_2_O (Fresenius). DNA concentrations were measured using a NanoDrop 1000 (Thermo Scientific), which allowed calculation of the amount of eDNA and iDNA in the biofilm. The results are given by the ratio of eDNA to iDNA. Samples of eDNA and iDNA were also separated and visualized on agarose gels (0.8%).

### Growth kinetics

Growth kinetics were carried out in transparent 24-well plates (Greiner) in 1 ml culture volume using M9 minimal medium supplemented with glucose (0.2%) and Tris/HCl pH 8.0 (100 mM), to remain the buffer capacity even under conditions without inorganic phosphate (von Kruger *et al*., 1999). The respective strains were grown in a pre-culture for 24 h in M9 Tris glucose with aeration and shaking at 37°C. Cells derived from the pre-cultures were washed with M9 Tris glucose without inorganic phosphate and adjusted to OD_600_ = 0.03 with either M9 Tris glucose or M9 Tris glucose without inorganic phosphate (0 mM) supplemented with 2.5 mg ml^−1^ herring sperm DNA as phosphate source. OD_600_ was monitored every 30 min in the FLUOstar OMEGA plate reader (BMG Labtech) at 37°C with shaking.

### Detachment assay

The detachment assay was performed as previously published (Zhu and Mekalanos, 2003; Liu *et al*., 2007), but with following modifications. An overnight culture of the respective strain was diluted 1:150 in LB broth. A borosilicate glass tube was inoculated with 1.5 ml of the resulting dilution and biofilm was allowed to form for 40 h under static conditions at RT. Glass tubes were subsequently rinsed two times with LB and 1.6 ml spent LB (obtained from a 40 h old biofilm culture of the respective strain) was added. One hundred microlitres was directly taken as sample T_o_ reflecting the original cfu in the supernatant, which result either from residual planktonic cells or mechanical disturbance of the biofilm due to addition of spent LB. After 3 h incubation at RT another sample from the supernatant was taken reflecting time point T_d_, which contains the cells from T_o_ and additionally cells that have detached during the 3 h incubation. The remaining biofilm was dispersed as described above in 2 ml fresh LB broth. This sample, called T_b_, reflects the cells remaining in the biofilm.

Appropriate dilutions were plated on LB plates and incubated at 37°C over night. Before plating, each sample was rigorously mixed by vortexing and treated in an ultrasonic water bath to disperse biofilms to avoid biofilm clumps masking the real cfu titre. Complete dispersion was confirmed by microscopy. The detachment rate was defined by the percentage of detached cells within the 3 h period compared with the whole cfu present in the original biofilm and calculated as follows: (cfu T**_d_** – cfu T**_o_**)/(cfu T**_d_** + cfu T**_o_** + cfu T**_b_**) × 100.

### Competition experiments

Competition experiments and mouse experiments were performed as previously described (Schild *et al*., 2007; Moisi *et al*., 2009). For competition experiments 5- to 6-day-old C57Bl/6 mice were used as well as LB broth as an *in vitro* control. Mice were separated from their dams 1 h before infection. Subsequently, they were anaesthetized by inhalation of isoflurane gas and then inoculated by oral gavage with 50 µl of the respective inoculum. To prepare the inocula, the respective biofilm-derived cells (*lacZ*^+^) and planktonic cells (*lacZ*^-^) were mixed at 1:1 ratios in LB broth before inoculation. To determine the input ratios and bacterial titres the inocula were plated on LB plates containing Xgal. Before plating each sample was rigorously mixed by vortexing and treated in an ultrasonic water bath to disperse biofilms to avoid biofilm clumps masking the real cfu as described above. Complete dispersion was confirmed by microscopy. The final titres were approximately 10^5^ cfu per mouse. The inoculated mice were kept away from their dams for 24 h. At 24 h post inoculation, mice were euthanized, the small bowel from each mouse was removed by dissection and mechanically homogenized in 1 ml LB broth with 30% glycerol. Appropriate dilutions starting with 100 µl of the homogenate were plated on LB/Sm/Xgal plates. The competition index is calculated as the blue/white ratio of the output normalized to the blue/white ratio of the input. Additionally the recovered cfu per small bowel of the planktonic and biofilm derived cells of the wild type and *ΔdnsΔxds* mutant are shown separately in Fig. S10.

To obtain biofilm-derived cell samples, biofilms were allowed to form under static conditions in 1.5 ml LB broth in borosilicate glass tubes for 48 h at RT. Biofilms were rinsed with LB broth, removed mechanically from the glass tubes and adjusted in 1.6 ml fresh LB. For all competition experiments, three independently grown biofilms were formed in parallel and pooled. Presence of uniform pieces of biofilm clumps with approximately 50–100 cells was confirmed by microscopy (Fig. S9). For planktonic cell sample preparation the respective strain was grown over night (16 h) in LB Sm broth and then adjusted to the desired concentration.

### Statistical analysis

Data were analysed using the Mann–Whitney *U*-test in the case of single comparisons or by a Kruskal–Wallis test followed by *post hoc* Dunn's multiple comparisons. Differences were considered significant for *P* values of < 0.05.
