# Supplementary material for: Extracellular nucleases and extracellular DNA play important roles in Vibrio cholerae biofilm formation
Source: Mol Microbiol. 2011 Oct 27;82(4):1015–37. doi: 10.1111/j.1365-2958.2011.07867.x (PMC3212620; doi:10.1111/j.1365-2958.2011.07867.x)
Supplement: Supplementary file 1 [file mmi0082-1015-SD1.pdf]

Extracellular nucleases and extracellular DNA play important roles in *Vibrio cholerae* biofilm formation

Andrea Seper[1], Vera H. I. Fengler[1], Sandro Roier[1], Heimo Wolinski[1], Sepp D. Kohlwein[1], Anne L. Bishop[2], Andrew Camilli[2], Joachim Reidl[1] and Stefan Schild[1*]

[1]Institut fuer Molekulare Biowissenschaften, Karl-Franzens-Universitaet Graz, Humboldtstrasse 50, 8010 Graz, Austria

[2]Howard Hughes Medical Institute and the Department of Molecular Biology and Microbiology, Tufts University School of Medicine, Boston, MA 02111, U.S.A.

(Running title: nucleases of *Vibrio cholerae*)

[*]Corresponding author: Mailing address: Institut fuer Molekulare Biowissenschaften, Karl-Franzens-Universitaet Graz, Humboldtstrasse 50, 8010 Graz. Phone: ++43 (0)316 380-1970. Fax: ++43 (0)316 380-9019. E-mail: stefan.schild@uni-graz.at

SUPPLEMENTAL DATA

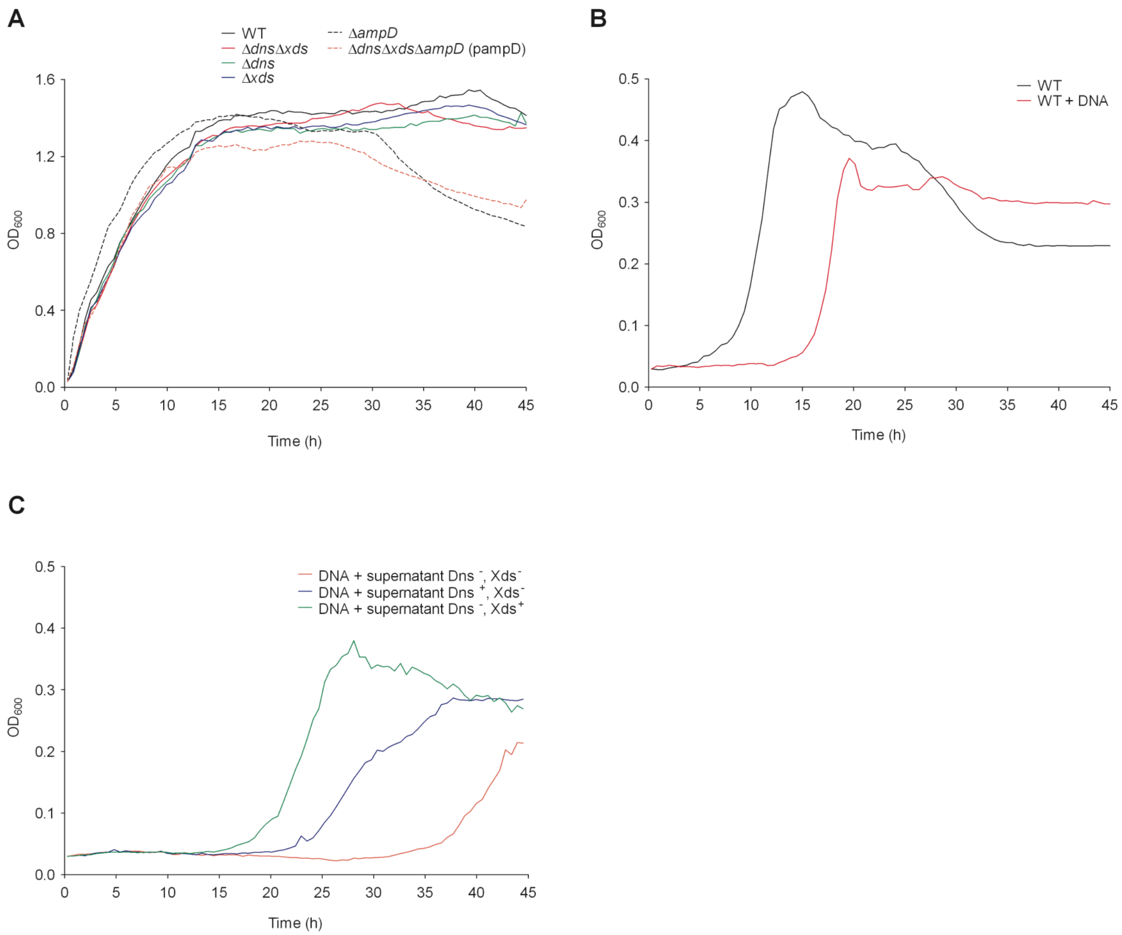

Fig. S1. Growth kinetics of the wild type, deletion mutants and complementation strains

A. Shown are the growth kinetics of wild type (black), Δ*dns*Δ*xds* (red), Δ*dns* (green), Δ*xds* (blue), Δ*ampD* (dashed black) and Δ*dns*Δ*xds*Δ*ampD* (pampD) (dashed red) monitored in LB broth. Values represent medians from at least four independent measurements.

B. Shown are the growth kinetics of wild type in M9 glucose (black) or in M9 glucose supplemented with 10 mg/ml herring sperm DNA (red). Values represent medians from at least four independent measurements.

C. Shown are growth kinetics of the double mutant under phosphate limiting conditions in M9 Tris glucose supplemented with pre-treated herring sperm DNA (2.5 mg/ml) as a phosphate source. Briefly, the DNA was incubated for 24 h in filter-sterilzed supernatants obtained from cultures of Δ*dns*Δ*xds* pMMB (red), Δ*dns*Δ*xds* pdns (blue) or Δ*dns*Δ*xds* pxds (green) prior to its

use as a source of phosphate in growth experiments using the double mutant. The supernatants were collected from cultures grown to an OD_{600} of ~ 0.2 in minimal media M9 with low phosphate concentrations (6.5 mM) supplemented with 0.2 % maltose as carbon source and 1 mM IPTG to allow induction of the respective nuclease. Thus, the supernatant of Δ*dns*Δ*xds* pdns and Δ*dns*Δ*xds* pxds contained Dns or Xds activity, respectively. The supernatant of Δ*dns*Δ*xds* pMMB lacking Dns and Xds activity served as a control.

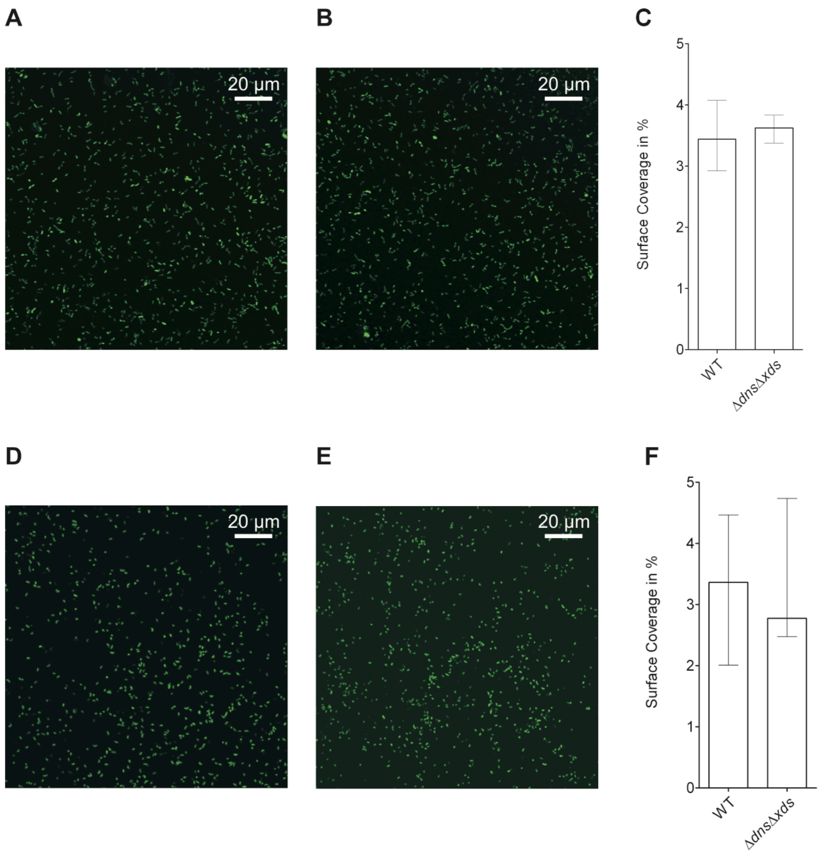

Fig. S2. Biofilms of the wild type and Δ*dns*Δ*xds* mutant at an early biofilm stage (monolayer).

Shown are confocal laser scanning microscopy images of wild type (A and D) and Δ*dns*Δ*xds* mutant (B and E) biofilms after incubation for 2 h in flow cell chambers supplied with LB (A - C) or 2% LB broth (D - F). Biofilms were stained with SYTO 9 fluorescent nucleic acid stain. Micrographs represent a single optical section comparing the attachment and surface coverage. The median surface coverage of the wild type and Δ*dns*Δ*xds* mutant was determined by the COMSTAT software for the incubation with LB (C) and 2% LB broth (F). For each strain at least six micrographs from three independent experiments were analyzed. The error bars indicate the interquartile range.

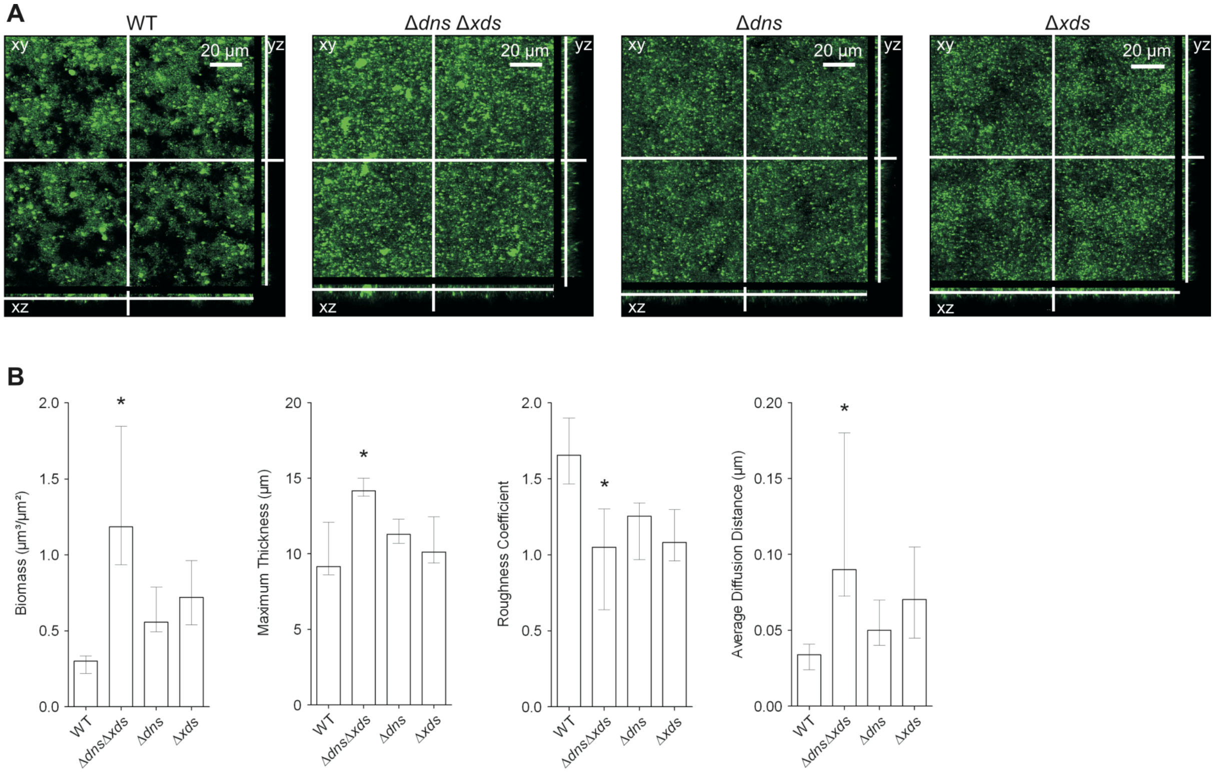

Fig. S3. Biofilm architecture using 50-fold diluted (2%) LB broth.

(A) Shown are confocal laser scanning microscopy images of the wild type, Δ*dns*Δ*xds*, Δ*dns* and Δ*xds* mutant biofilms as horizontal (xy) and vertical (xz and yz) projections (large and side panels, respectively). Biofilms were allowed to form for 24 h in flow cell chambers supplied with 2% LB and stained with SYTO 9 fluorescent nucleic acid stain. Images represent selected single optical sections through the acquired three-dimensional data sets.

(B) Image stacks of the wild type and mutant biofilms were analyzed for the biomass, the maximum thickness, the roughness coefficient and the average diffusion distance using the COMSTAT software. Shown are the medians of at least six image stacks from three independent experiments for each strain. The error bars indicate the interquartile range. Significant differences (* $P<0.05$) of structural parameters are indicated for the multiple comparisons of the deletion mutants with the wild type.

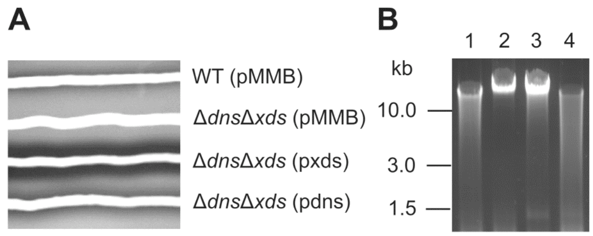

Fig. S4. Detection of extracellular nuclease activity

A. Indicated strains were grown 24 h at 37°C on BBL™ DNAse test agar (BD) according to the manufacturer's instructions. Flooding with 1 N HCl precipitates the remaining DNA and allows visualization of nuclease activity by zones of clearing.

B. Shown is an agarose gel visualizing the degradation of *V. cholerae* chromosomal DNA after incubation for 6 h with supernatants derived from cultures of wild type (pMMB) (lane 1), Δ*dns*Δ*xds* (pMMB) (lane 2), Δ*dns*Δ*xds* (pxds) (lane 3) or Δ*dns*Δ*xds* (pdns) (lane 4). For details see Experimental Procedures/ DNAse activity assays.

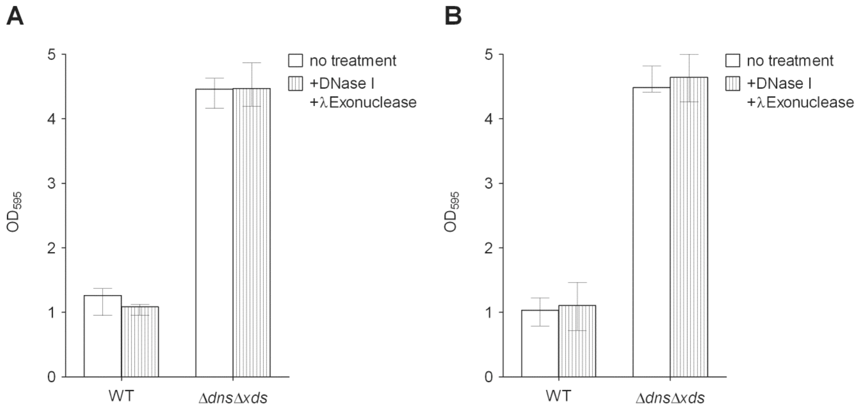

Fig. S5. Analysis of nuclease sensitivity of *V. cholerae* biofilms at late stages.

Biofilms of wild type (WT) and Δ*dns*Δ*xds* mutant grown under static conditions for 69 h (A) or 65 h (B) were treated with a combination of DNase I and λExonuclease (horizontally shaded bars) for 3 h (A) or 7 h (B). Thus, the overall time of biofilm formation was 72 h. Incubation with nuclease buffer alone was used as control condition reflecting biofilms without treatment (open bars). Biofilm mass remaining after nuclease treatment was quantified by crystal violet staining. Shown are the medians from at least 6 independent measurements. The error bars indicate the interquartile range. No significant differences compared to untreated biofilms of the wild type or Δ*dns*Δ*xds* mutant were detected (* $P<0.05$).

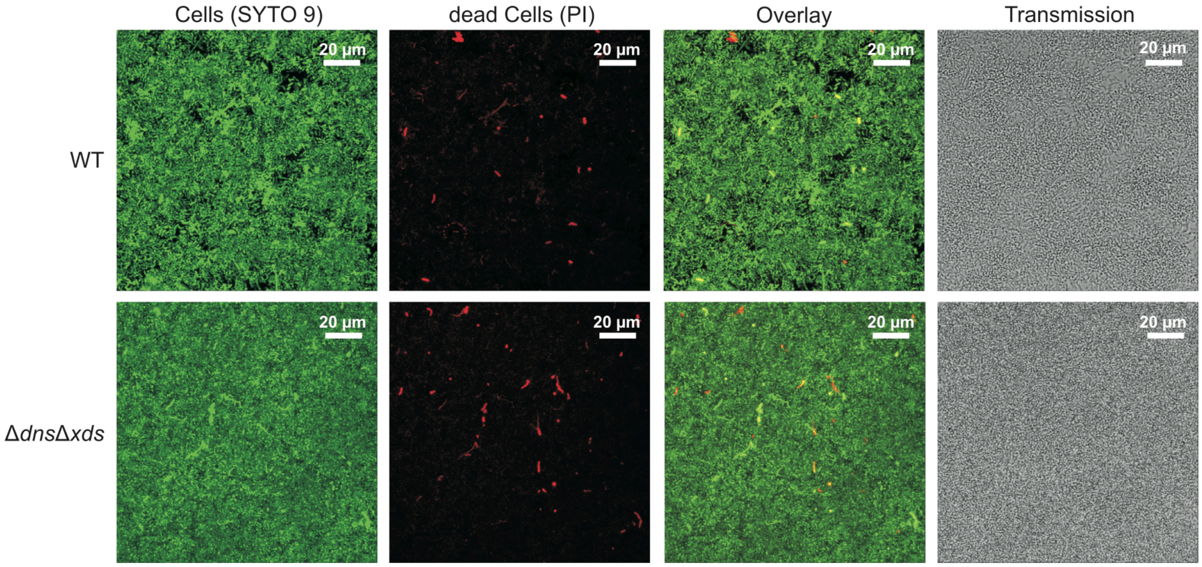

Fig. S6. Impact of nuclease deletion on cell death in *V. cholerae* biofilms.

Live/Dead staining of wild type (WT) and Δ*dns*Δ*xds* mutant biofilms grown in flow cells for 9 h as described in Experimental Procedures. Live cells appear green due to SYTO 9 staining and dead cells appear red due to Propidium Iodide (PI) staining. Live/Dead BacLight Bacterial Viability kit (Invitrogen) was used according to the manufacturer's manual. Every channel was inoculated with approximately 250 µl of the fluorescent dye solution (SYTO 9 and PI) and stained at RT for 20 min. Images of biofilms were recorded by confocal laser scanning microscopy and represent maximum-intensity projections.

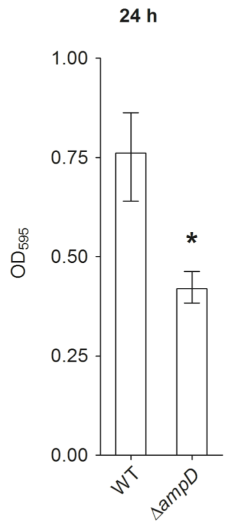

Fig. S7. Deletion of *ampD* results in decreased biofilm formation.
Biofilms of the wild type (WT) and the Δ*ampD* mutant were quantified after 24 h. The biofilm formation capacity was assayed under static conditions by crystal violet staining and subsequent determination of the OD_{595}. Shown are the medians from at least 8 independent measurements. The error bars indicate the interquartile range. Significant differences (* P<0.05) are indicated for the comparison of wild type and Δ*ampD* mutant.

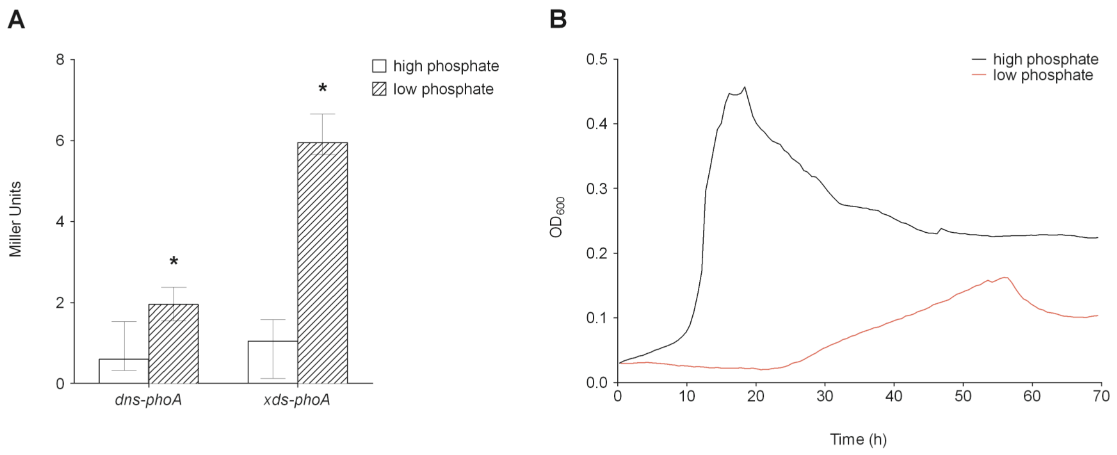

Fig. S8. Expression of *dns* and *xds* under conditions of phosphate limitation.

A. Shown are the alkaline phosphatase activities (in Miller Units) of wild type with either a chromosomal *dns-phoA* or *xds-phoA* transcriptional fusion. Cultures were grown with aeration until they reached an OD_{600} of ~ 0.2 under conditions of high phosphate (M9 Tris glucose, inorganic 65 mM phosphate) or low phosphate (M9 Tris glucose, inorganic 6.5 mM phosphate). Shown are the medians from at least six independent measurements. The error bars indicate the interquartile range. The activities of both *phoA*-fusions are significantly increased under low phosphate conditions (* $P<0.05$).

B. Shown are the growth kinetics of wild type under conditions of high phosphate (M9 Tris glucose, inorganic 65 mM phosphate) or low phosphate (M9 Tris glucose, inorganic 6.5 mM phosphate). Values represent medians from at least six independent measurements.

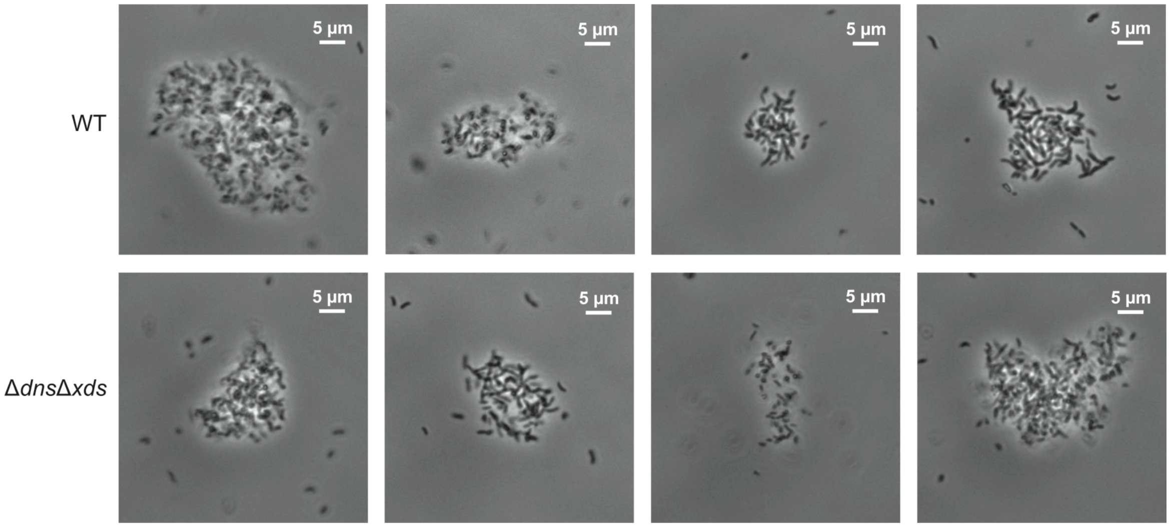

Fig. S9. Visualization of dispersed biofilm clumps.

Shown are representative phase-contrast micrographs of biofilm clumps of the wild type (WT) and the Δ*dns*Δ*xds* mutant used for the in vivo colonization. Biofilms were grown under static conditions for 48 h and then dispersed as described in Experimental Procedures. Images of biofilms were recorded using a Leitz Laborlux D microscope (400x magnification) equipped with a video camera system (Visitron systems).

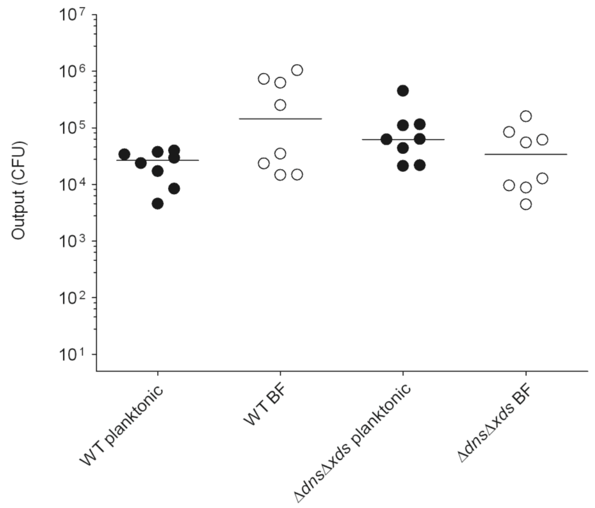

Fig. S10. In vivo colonization of planktonic and biofilm derived cells.

Shown are the recovered CFU per small bowel for planktonic and biofilm derived cells of the wild type (WT) and the Δ*dns*Δ*xds* mutant. The CFU correspond to the competitive indices (CI) shown in Fig. 9. Each circle represents the recovered CFU from one mouse. Horizontal lines indicate medians for each data set.
